# Harnessing microbial metabolism and nutrition to target the gut–mammary axis for better livestock production and health

**DOI:** 10.1002/imo2.70114

**Published:** 2026-07-09

**Authors:** Aolong Zhou, Cheng Guo, Mahmoud M. Abdelsattar, Ahmad R. Musa, Mequanint Biks, Abdullahi A. Musa, Shahnaz Rahman, Abera Teshome Aleli, Pei Zhong, Jiawei Zhang, Junwei Cui, Xiyan Kou, Qingyuan Yu, Caijuan Yue, Qin Li, Dan Zhang, Zixuan Peng, Ao Ren, Ziqi Tian, Ziqi Liu, Yaona Zhang, Lili Zhang, Dina Karmallah, Naifeng Zhang, Tao Ma, Yan Tu, Qiyu Diao, Guijie Zhang, Xiaofeng Xu, Yanliang Bi

**Affiliations:** ^1^ College of Animal Science and Technology Ningxia University Yinchuan China; ^2^ Key Laboratory of Feed Biotechnology of the Ministry of Agriculture and Rural Affairs, Institute of Feed Research Chinese Academy of Agricultural Sciences Beijing China; ^3^ Department of Animal and Poultry Production, Faculty of Agriculture Qena University Qena Egypt; ^4^ Department of Animal Science Federal University, Gashua Gashua Yobe State Nigeria; ^5^ Department of Animal Sciences Debre Tabor University Debre Tabor Ethiopia; ^6^ Department of Biochemistry and Molecular Biology Gono University Savar Dhaka Bangladesh; ^7^ Ambo University, Guder Mamo Mezemir Campus Ambo Ethiopia; ^8^ Institute of Animal Science Ningxia Academy of Agricultural and Forestry Sciences Yinchuan China

**Keywords:** gut microbiota, gut–mammary axis, mammary health, maternal‐offspring transmission, precision nutrition

## Abstract

The gut‐mammary axis orchestrates bidirectional crosstalk linking intestinal homeostasis and mammary function through integrated neural, endocrine, and immune pathways. Dysregulation driven by gut microbiota dysbiosis or metabolic dysfunction compromises lactation performance and induces mastitis via pathogen translocation and epigenetic reprogramming. This review systematically discusses the molecular mechanisms underlying microbial metabolite‐mediated mammary development and lactation efficiency, evaluates the impact of gut‐derived pathogens on mammary barrier integrity and inflammatory cascades, explores maternal offspring microbiome transmission for neonatal immunity programming, and proposes precise nutritional strategies targeting microbial immune metabolic networks. Our synthesis of previous research findings aimed to optimize animal productivity, prevent mammary gland disorders, and provide insights for the advancement of the livestock industry.

## Highlights

- The gut‐mammary axis links intestinal ecology and nutrition to mammary function via neural, endocrine, and immune pathways.
- Nutritional interventions improve milk quality, relieve inflammation, and enhance lactation performance through microbial metabolite signaling and immune cell trafficking.
- Maternal gut microbes vertically transmit through milk to boost offspring immunity and growth.

## Graphical abstract

The gut–mammary axis integrates intestinal health with mammary function through neural, endocrine, and immune pathways. Nutritional interventions (probiotics, prebiotics, metabolites) improve gut microecology, generating microbial signals that travel via the axis to the mammary gland. There, they relieve inflammation, enhance milk quality and lactation, and boost offspring growth. This framework highlights precision nutrition as a strategy to modulate the gut–mammary axis for sustainable livestock production.

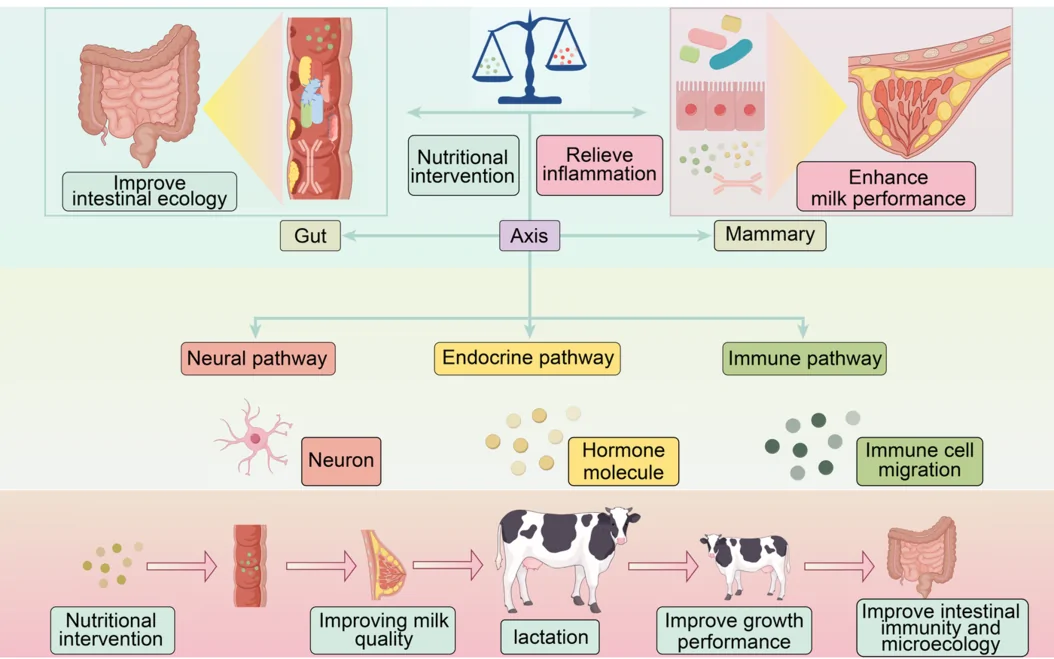

## 1 INTRODUCTION

Recently, the introduction of the gut–brain, gut–lung, gut–liver, and gut–kidney axes has triggered research into the mechanisms underlying these interactions [1, 2, 3, 4]. Unlike these anatomically connected axes, the gut–mammary axis represents a functional rather than an anatomical conduit that integrates intestinal and mammary functions through systemic circulation, neuroendocrine signaling, and immune cell trafficking. This distinction is critical because the mammary gland lacks direct anatomical connections with the gastrointestinal tract; instead, communication occurs via blood‐borne microbial metabolites, hormones, and immune cells that migrate from the gut‐associated lymphoid tissues to the mammary gland. The gut–mammary axis has been studied across multiple mammalian species, serving distinct roles in the discovery to application pipeline. Rodents (mice and rats) are the most widely used models for mechanistic studies owing to their genetic tractability, short reproductive cycles, and well‐characterized immune systems. However, their gastrointestinal tract is simple, and they possess five pairs of mammary glands with relatively low milk output. Pigs serve as an important translational bridge between rodents and production livestock. They share physiological similarities with humans and other large mammals, have a monogastric digestive system similar to that of rodents, and their mammary gland architecture and lactation demand more closely resemble those of production animals. Ruminants are the primary target species for production‐oriented applications. Their forestomach enables extensive microbial fermentation that profoundly alters the metabolism of dietary compounds before they reach the mammary glands. Ruminants also have the highest lactation demand and a distinct mammary gland structure. Understanding these anatomical and physiological differences is essential for interpreting cross‐species data and translating mechanistic insights from model organisms into effective livestock management interventions.

The “gut‐mammary axis” constitutes a complex regulatory network connecting the intestine and mammary glands, which has attracted considerable attention because of its key role in animal production and health. It dynamically integrates intestinal and mammary functions through multidimensional pathways, such as neural signaling, endocrine regulation, and immune interactions. First, the gut microbiome and its metabolites are transported to the mammary gland via the circulatory system, thereby directly regulating milk component synthesis, inflammatory responses, and pathogen defenses. In addition, hormones regulating the mammary glands (e.g., prolactin) and immunologically active substances (e.g., secretory immunoglobulin A (sIgA) can regulate intestinal barrier function and microbiome homeostasis through a feedback mechanism [5, 6, 7]. These findings expand the theoretical framework that represents the pathogenesis of mammary disease and also provide a scientific basis for optimizing gut‐mammary axis function through nutritional interventions (e.g., probiotics and functional metabolites). In this review, we systematically discuss the neural, endocrine, and immune regulatory mechanisms underlying the gut‐mammary axis. We further explore its role in regulating animal performance through nutrient uptake, metabolic reprogramming, and immune homeostasis maintenance. This synthesis aims to provide theoretical support for the precise management of mammary gland health and sustainable livestock production.

While previous reviews have provided valuable insights into the connection between the gut microbiota and mammary glands, several important knowledge gaps remain. First, most existing literature focuses on human or rodent models, with limited systematic integration of findings across livestock species (e.g., dairy cows, sows, and goats) that are central to animal production. Second, the mechanistic crosstalk among the neural, endocrine, and immune pathways has been discussed separately, but a holistic framework that captures the synergistic regulation of the gut‐mammary axis is lacking. Third, although nutritional interventions (e.g., probiotics, prebiotics, and functional metabolites) have shown promise, a comprehensive synthesis of their specific effects on lactation performance, mammary health, and offspring development in livestock has never been conducted. Fourth, the bidirectional nature of this axis, particularly how mammary‐derived factors feedback to modulate intestinal function, remains underexplored in production settings. This review addresses these gaps by providing a species focused synthesis that prioritizes relevant evidence from livestock, integrating the neuro endocrine immune network into a unified conceptual framework, and critically evaluating precise nutritional strategies that target the gut–mammary axis. Furthermore, we highlight the translational potential of this axis for improving animal productivity and health, with particular emphasis on maternal offspring microbiome transmission and sustainable livestock production.

Unlike previous reviews that focused mainly on human or rodent models or isolated mechanisms, this review offers three unique contributions: a species focused synthesis of livestock relevant evidence, a direct link between neuro endocrine immune pathways and practical nutritional strategies, and a critical assessment of translational barriers such as species specific physiology and industrial scalability. Within this framework, the term precision nutrition refers to targeted strategies that optimize the gut–mammary axis to improve lactation, mammary health, and offspring development. These strategies include stage specific maternal nutrition, microbiome guided supplementation, metabolite based interventions, and farm level decision support systems using real time biomarkers. The following sections critically evaluate each approach, focusing on species specific feasibility and translational barriers.

## 2 PHYSIOLOGICAL BASIS OF THE GUT–MAMMARY AXIS IN ANIMALS

### Gut health affects mammary health

The mammalian intestinal tract, which is the longest digestive organ in animals, has a complex multifunctional structure. The inner wall is lined with folds and villi that substantially increase the surface area to facilitate nutrient absorption. The mucosal layer of the intestinal tract is rich in various digestive enzymes and nutrient absorption related transport proteins that efficiently break down food into small molecules entering the bloodstream or lymphatic system through active transport or passive diffusion [8]. Moreover, the intestinal tract plays a crucial role in immune defense. The intestinal mucosal barrier, gut microbiota, and gut associated lymphoid tissues collectively form a robust line of defence that protects the host from external pathogens [9].

The gut microbiota comprises complex communities of bacteria, fungi, viruses, and archaea residing in animal intestines [10]. They provide energy to the host by breaking down dietary fibers to produce short‐chain fatty acids (SCFAs), synthesize essential nutrients such as vitamins K and B, protect host health by regulating the immune response, and maintain intestinal barrier integrity. They also participate in metabolic regulation, neural signaling, and systemic inflammatory homeostasis [11, 12, 13]. Therefore, intestinal dysbiosis may contribute to the onset and progression of mammary diseases. Disruptions of the intestinal microbiome might simultaneously or subsequently affect the probiotic environment, which can cause probiotics to lose their “dominant” role in the tumor environment. This negatively affects the control or inhibition of mammary tumor cell growth and even accelerates tumor cell growth and promotes angiogenesis, indirectly contributing to tumor metastasis [14, 15, 16]. Although mammary cancer is not a primary production issue in livestock, the underlying signaling pathways are evolutionarily conserved and directly regulate lactation performance, mammary epithelial cell turnover, and inflammatory responses, thereby providing mechanistic insights applicable to mastitis prevention and milk production. In addition, intestinal dysbiosis undermines the integrity of the intestinal barrier and promotes the migration of specific pathogenic bacteria through the circulatory system to the mammary glands. He et al. [17] demonstrated that enterogenic *Aeromonas* species can migrate from the intestines to the mammary glands and may contribute to the development of mastitis in mice. In contrast, gut health and balanced microbiota exert positive effects on mammary health. According to recent studies, probiotic supplementation (e.g., *Lactobacillus suis R389* and *Lactobacillus reuteri*) protects against mammary tumor development in mouse models [18, 19]. In addition, Kassayova et al. [20] showed that *Lactobacillus plantarum LS/07* or dietary fiber supplementation ameliorated mammary carcinogenesis in rats. These findings indicate that regulating intestinal health can positively impact mammary health. Although the endpoints used in rodent mammary tumor studies are not directly applicable to livestock, these experiments reveal conserved immunomodulatory pathways (e.g., NF‐κB, STAT3) that also regulate mammary gland inflammation and immune defense.

### Mammary function affects intestinal function

The mammary gland, a unique organ in mammals, undergoes development and lactation, and is regulated by numerous hormones and growth factors. During animal growth and development, the mammary gland undergoes multiple morphological and functional changes, gradually developing from an undifferentiated state at the embryonic stage to a mature gland capable of lactation [21]. Lactation involves the uptake, synthesis, and secretion of blood‐derived nutrients by mammary epithelial cells, a process regulated by hormones such as prolactin and insulin‐like growth factor [22]. As a major secretory organ, the mammary gland's lactation function is regulated by intestinal nutrient supply, and it also exerts feedback effects on intestinal nutrient absorption and metabolism [23]. During lactation in particular, the high nutrient demand of the mammary glands stimulates the intestines to enhance nutrient absorption efficiency [24]. Mammary milk is the “bridge” between the mother's mammary glands and the infant's intestines and provides nutrients and immune components that are crucial for defense against bacterial infections. Immunoglobulins and antimicrobial peptides in milk enhance the mucosal barrier function of the newborn intestine and promote colonization by beneficial bacteria such as *Lactobacillus spp*., thereby fostering a healthy gut microbiome [25, 26, 27].

## 3 MECHANISMS OF GUT–MAMMARY AXIS REGULATION IN ANIMALS

### Connecting pathways of the gut–mammary axis: Neurologic, endocrine, and immune

The nervous, endocrine, and immune systems interact dynamically to integrate the functions of the gut and mammary glands [28]. First, the neural pathway primarily transmits sensory information from the gut to the central nervous system (CNS) via the vagus nerve (VN). Subsequently, the CNS regulates mammary gland development and lactation through pathways, such as the hypothalamic pituitary axis [29]. Acetylcholine released from VN endings directly acts on cholinergic receptors in mammary epithelial cells to promote milk protein synthesis [30]. Simultaneously, the hypothalamic pituitary axis dynamically adapts to mammary gland lactation demands (e.g., suckling stimulation in young animals) by regulating the prolactin secretion cycle [31]. These components of the endocrine pathway link the gut to the mammary glands through hormones that circulate in the blood. For example, hormones secreted by the intestines, such as glucagon‐like peptide‐1 (GLP‐1) and peptide YY (PYY), promote the efficient utilization of glucose by mammary epithelial cells by lowering blood glucose and insulin levels, which, in turn, affect mammary gland development and lactation function. Pituitary hormones, such as prolactin, also act on the intestinal tract to regulate intestinal digestion, absorption, and immune function [32, 33]. The immune system regulates inflammation, barrier repair, and immune memory signals between the gut and mammary glands through the directional migration of intestinal microbial metabolites (e.g., SCFAs), immune cells (e.g., memory B cells), and the remote interactions of immune molecules (e.g., sIgA and IL‐10) [34]. All of these immune interactions collectively maintain physiological homeostasis and improve animal performance.

### Mechanisms of neuromodulation

The VN, an important part of the autonomic nervous system, serves as a bridge between the gut and the CNS of mammals (Figure 1). The VN senses intestinal stimuli (e.g., nutrient absorption and changes in the gut microbiota) and transmits this information to the brain via neural reflexes, influencing mammary gland development and lactation [35]. The VN regulates the proliferation, differentiation, and secretory activity of mammary epithelial cells via neurotransmitters, such as acetylcholine and norepinephrine. This mechanism enables the mammary glands to respond adaptively to the overall needs of the organism and to environmental changes [36]. In addition to long‐distance vagal regulation, localized neural reflexes and regulatory mechanisms link the gut and mammary glands. These localized neural networks include nerve endings, plexuses, and neurons connecting the gut to the mammary glands [37]. These networks detect local environmental changes. For instance, increased intramammary pressure is detected by local nerve endings, which trigger nerve reflexes that promote mammary duct contraction and milk ejection [38]. Similarly, when nutrients are abundant in the gut, local neural networks enhance nutrient absorption and delivery to support lactation by regulating intestinal muscle contraction, relaxation (motility), and digestive enzyme secretion [39]. Local neurological reflexes and regulatory mechanisms help maintain the dynamic balance between the gut and mammary glands, and adapt to external environments. During lactation, suckling by pups stimulates the mammary glands as the demand for milk increases, which rapidly increases the amount of milk produced to meet the needs of the pups through localized nerve reflexes [40]. Simultaneously, the intestines boost their digestive and absorptive functions to ensure that the mammary glands are supplied with sufficient nutrients to support lactation [41]. This synergy allows animals to maintain efficient lactation in complex environments.

**Figure 1:**
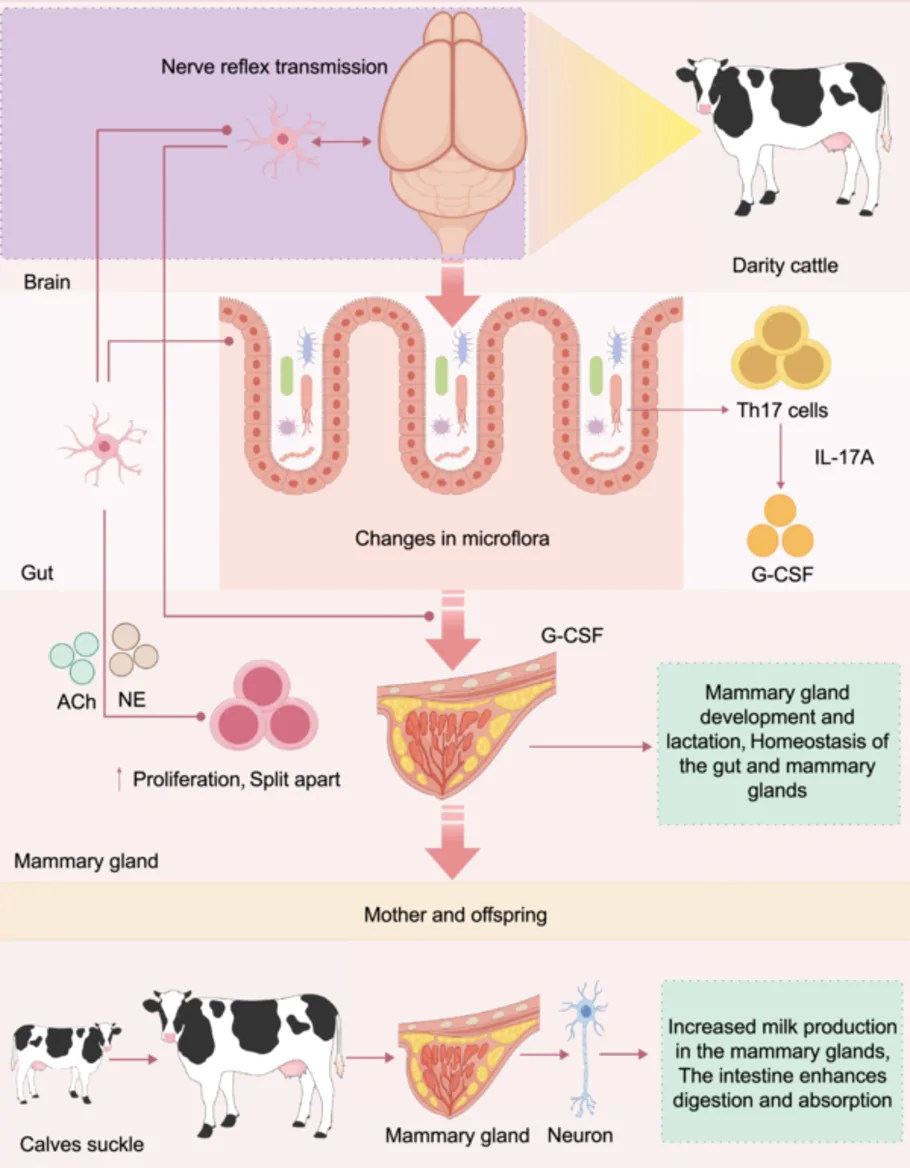
Neuromodulatory mechanisms of the gut–mammary axis. Multifaceted regulatory network of the gut–mammary axis in dairy cattle: neuro‐immuno‐microbial crosstalk mediating mammary development, lactation homeostasis, and maternal‐offspring nutrient symbiosis. ACh, acetylcholine; G‐CSF, Granulocyte colony‐stimulating factor; IL‐17, Interleukin 17; NE, Norepinephrine; Th17, Helper T cell 17.

### Endocrine regulatory mechanisms

Hormones play a crucial role in regulating the gut–mammary axis, and various hormones transmit messages between the gut and mammary glands through the bloodstream, coordinating the physiological functions of both organs (Figure 2). For example, GLP‐1 not only promotes glucose metabolism by enhancing insulin sensitivity but also acts on mammary epithelial cells to inhibit mTORC1 activity by activating the AMPK signaling pathway in human breast cancer cell lines. The AMPK signaling pathway, in turn, blocks mammary cancer cell cycle progression, reducing mammary cancer cell proliferation and migration, and is associated with a lower incidence of mammary cancer in experimental models [42, 43, 44]. While mammary carcinoma is not a primary concern in livestock production, studies highlight the GLP‐1‐AMPK‐mTORC1 signaling axis, which regulates milk protein synthesis and mammary gland metabolism (see Section 4.1). This suggests that gut hormones simultaneously regulate gut metabolism and mammary gland function via the endocrine pathway and that the hormones secreted by the intestines indirectly regulate the lactation function of the mammary glands by influencing appetite, energy metabolism, and other functions [45]. For example, GLP‐1 enhances insulin secretion and sensitivity and promotes glucose utilization and storage, thus increasing nutrient uptake efficiency and utilization by mammary epithelial cells [46]. In murine mammary epithelial cells, Peptide YY (PYY) inhibits the NF‐κB pathway by binding to the mammary Y2 receptor to alleviate the mammary inflammatory response. Concurrently, it indirectly reduces mammary lipid synthesis load and affects the oxidative stability of mammary lipids by reducing energy intake through central appetite regulation [47, 48, 49]. In contrast, in humans and rodents, mammary‐derived leptin is a multi‐targeted regulator of intestinal mucosal barrier integrity, immune homeostasis, and microbiota host interactions through autocrine and paracrine pathways. An imbalance in leptin signaling pathways is associated with the pathogenesis of inflammatory bowel disease and metabolic syndrome, providing a new theoretical basis for targeted intervention [50, 51, 52, 53]. In neonatal mice, prolactin secreted by the pituitary gland promotes milk synthesis and acts on the intestines through blood circulation, regulating the expression of glucose transporter proteins in intestinal epithelial cells and affecting the efficiency of intestinal absorption of carbohydrates [54]. Studies indicate that endocrine regulation of the gut–mammary axis achieves a dynamic balance between gut and mammary functions through hormone‐mediated metabolic reprogramming, signaling pathway intervention, and immune metabolic interactions. Additionally, the multi‐targeted and bi‐directional regulation of the gut–mammary axis provides novel molecular targets and intervention strategies for the prevention and treatment of metabolic diseases and mammary cancer.

**Figure 2:**
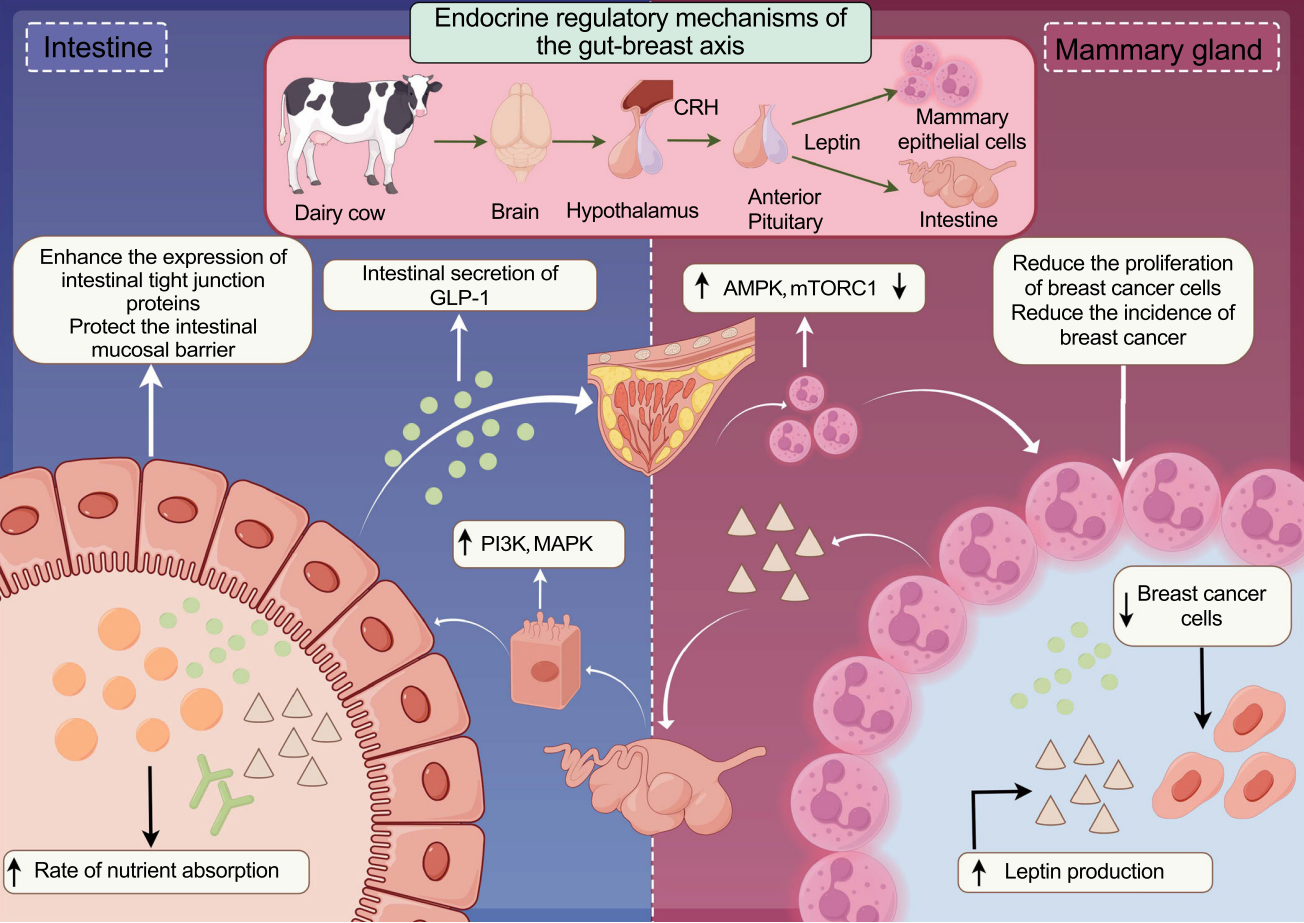
Endocrine regulatory mechanisms of the gut–mammary axis. The gut activates the AMPK/mTORC1 pathway through short‐chain fatty acids (SCFAs), inhibiting excessive lipid deposition in the mammary gland and promoting mammary protein synthesis via the GLP‐1/PI3K‐Akt signaling pathway. Leptin secreted by the mammary gland maintains barrier integrity by enhancing the expression of intestinal tight junction proteins (such as occludin), whereas prolactin upregulates the intestinal glucose transporter (SGLT1) to enhance nutrient absorption efficiency. Key pathways (MAPK/ERK, JAK2/STAT5) synergistically optimize milk composition and reduce disease risk by inhibiting mammary cancer cell metastasis and regulating mammary protein gene expression, ultimately achieving functional integration of metabolism, immunity, and lactation. CRH, adrenocorticotropic hormone‐releasing hormone. AMPK, AMP‐activated protein kinase; GLP‐1, glucagon‐like peptide‐1; mTORC1, mTOR complex 1; PI3K, phosphatidylinositol 3‐kinase; MAPK, mitogen‐activated protein kinases.

Notably, most of the endocrine mechanisms described above, particularly those involving GLP‐1, PYY, and leptin, were originally characterized in rodent or human models under non‐lactating or disease conditions. Whether these pathways operate with identical efficiency and regulatory dynamics in ruminants during metabolic stress under lactation remains largely unexplored. Ruminants possess a distinct digestive physiology (rumen fermentation) and face extreme nutrient demands that may alter hormone secretion patterns, receptor expression, or downstream signaling thresholds. Therefore, while the core signaling pathways (e.g., AMPK‐mTORC1, STAT5, and NF‐κB) are evolutionarily conserved, their quantitative contribution to lactation performance and mammary health in livestock requires direct validation in target species under production‐relevant conditions.

### Mechanisms of immunoregulation

Intestinal immune cells and molecules regulate local intestinal immune responses as well as the immune function of the mammary gland through the gut‐mammary axis (Figure 3). Intestinal immunogenesis depends on several factors, the most important being the relations between the immunogen and the intestinal immune system, and the influence of the intestinal environment [55]. For example, probiotics in the gut can reduce the incidence of intestinal inflammatory reactions and systemic immune responses by regulating intestinal microbiome balance, which is conducive to mammary gland health and the maintenance of lactation functions [56]. Milk also contains various immunologically active substances (e.g., immunoglobulins and lactoferrin) that enhance offspring immunity and promote growth and development [57]. Recently, modulation of beneficial gut microbiota abundance and diversity has been found to enhance immune regulation and ameliorate diffuse mammary hyperplasia in mice [58]. Therefore, the gut–mammary axis exerts an important influence on the immune function of animals. The immune pathways and regulatory mechanisms of the gut–mammary gland axis are shown in Figure 3. The gut to mammary immunization pathway begins with the intestinal epithelium, through which immunoglobulin A (IgA) enters the circulatory system and migrates to mammary tissues [7]. Memory B cells in the intestinal epithelium migrate through the circulatory system to mammary tissues [59]. In mammary tissues, secretory IgA (sIgA) facilitates immune cell migration through C–C chemokine receptor 10 (CCR10) interactions [60, 61]. sIgA cells secrete sIgA into milk via polyimmunoglobulin receptors on mammary epithelial cells [62], thus converting maternal intestinal immunity into passive neonatal immune protection, a process that is synergistically regulated by intestinal microbiome metabolites, immune signals, and the neuroendocrine system. Furthermore, modulating the composition of gut microbiota can enhance immune regulatory functions and improve mammary hyperplasia in experimental models. These findings are discussed in Section 4.2, drawing on the original literature.

**Figure 3:**
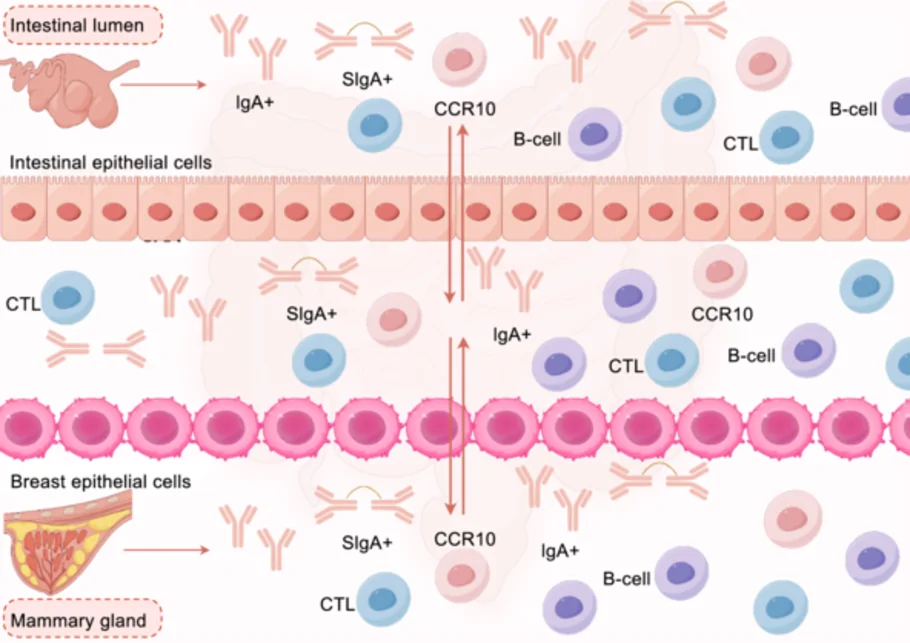
Immune pathways and regulatory mechanisms of the gut‐mammary axis. Gut‐mammary immune crosstalk: bidirectional ccr10+ lymphocyte trafficking, secretory iga/siga transport, and tissue‐specific immune surveillance in lactation and neonatal immunity. CTL, cytotoxic T lymphocytes; CCR10, Chemokine receptor.

#### Gut barrier immunity and microbial interactions

The gut microbiota is one of the largest microecosystems in the animal body, which is closely associated with the host immune system. Intestinal microbiota are involved in the regulation of immune function and inflammation by modifying the integrity of the intestinal mucosal barrier, activating and differentiating immune cells, and producing immune molecules [63]. Research indicates that dysbiosis of the human gut microbiota can lead to the disruption of intestinal barrier function and increased permeability. This triggers systemic inflammation and subclinical endotoxinemia and promotes the migration of pathogenic bacteria from the gut to the mammary glands, thereby inducing mastitis [64, 65]. Interactions between the intestinal microbiome and the mucosal immune system lead to an increase in potentially pathogenic gram negative intestinal bacteria and their associated metabolic changes, disrupting the intestinal epithelial immune barrier and increasing susceptibility to infection [66]. These processes affect gut health and directly or indirectly affect mammary gland health and function through the gut‐mammary axis. Probiotics can reduce intestinal inflammation and systemic immune responses by balancing the intestinal microbiome, thereby contributing to the maintenance of mammary gland health and lactation [67]. Therefore, gut microecology affects mammary health. In summary, the intestinal barrier serves as the first line of defense, integrating the physical, chemical, and immunological functions that are dynamically regulated by the gut microbiota. Disruption of this barrier not only compromises gut health but also predisposes the mammary gland to infection via the gut‐mammary axis. In addition to immune regulation, the gut microbiota influence mammary epithelial cell metabolism and milk protein synthesis. Specifically, intestinal microorganisms modulate the expression of milk protein genes and the activity of related transcription factors, thereby affecting the quantity and composition of milk proteins synthesized. These mechanisms, which involve microbial metabolite signaling and nutrient partitioning, are discussed in detail with supporting evidence in Section 4.1.1.

#### Immune cell trafficking along the gut‐mammary axis

The transmission of immune cells and molecules in the gut‐mammary axis is an important part of the immunoregulatory mechanism; they transmit information and play a regulatory role between the gut and mammary glands through blood or lymphatic circulation [68]. Macrophages, dendritic cells, and other immune cells phagocytose and process pathogens or antigenic substances in the intestinal tract and then enter the lymphatic or blood circulation to migrate to the mammary tissue, activating local immune cells in the mammary gland and triggering localized immune responses through antigen presentation and cytokine secretion [69]. Additionally, immune molecules, such as cytokines and chemokines, can influence the inflammatory response and lactation function of the mammary gland by regulating the activation and migration of immune cells [70]. These trafficking processes for immune cells and molecules defend against pathogen attack and the spread of infection and participate in regulating mammary physiological balance and productivity [71]. In addition, the inflammatory response is a defense mechanism of the body against stimuli such as injury or infection [72]. A moderate inflammatory response is essential for pathogen clearance and tissue repair within the gut–mammary axis [73]. However, excessive inflammation can damage the mammary tissues and affect lactation performance [74]. In summary, the directional migration of immune cells, particularly IgA+ plasma cells and memory B cells, from gut‐associated lymphoid tissues to the mammary gland is orchestrated by chemokine receptors such as CCR10 and their ligands. This trafficking mechanism ensures that maternal intestinal immunity is effectively transferred to the mammary gland and subsequently to their offspring via milk, thereby providing passive immune protection during early life.

## 4 REGULATION OF THE GUT–MAMMARY AXIS IN ANIMAL PRODUCTION

### Modulation of the gut–mammary axis affects animal milk performance

#### Influence of gut–mammary axis regulation on milk proteins

Interactions between the gut microbial community and the mammary glands offer significant promise in regulating milk quality and composition. As discussed in Section 3.3.1, the gut microbiota influences mammary functions through multiple pathways. Recent research indicates that gut microbiota can influence dairy cow lactation performance via the gut–mammary axis [75]. Intestinal microorganisms influence the metabolic processes of mammary cells and alter the expression levels of protein genes (e.g., *αS1‐casein* (*CSN1S1*) and *β‐lactoglobulin* (*BLG*) *genes*), thus affecting the amount of milk protein synthesized [76]. Moreover, gut microbes regulate the expression of transcription factors associated with protein synthesis in mammary cells, resulting in an increase in protein synthesis or changes in the ratio of their isoforms [77]. For instance, a controlled dose response trial in lactating dairy cows demonstrated that dietary inulin supplementation at a level of 200 g/d per cow significantly increased milk yield, milk protein content, and lactose production rate, while also modulating ruminal propionate concentrations. In the same dose finding study, inulin inclusion rates ranging from 100 to 400 g/d per cow consistently elevated milk protein and lactose yields, and the most pronounced effect on milk protein was observed at the 200 g/d dose [78, 79, 80]. These quantitative data provide a dose‐related reference for translating inulin supplementation into practical dairy feeding strategies. For inulin, although a 200 g/d dosage consistently improved milk protein content, the magnitude of the protein response varied by approximately 0.1–0.3 percentage points depending on basal diet and lactation stage. The positive effects of inulin on milk protein synthesis can be primarily attributed to the increased production of propionic acid, which acts as a key glucogenic precursor. Propionic acid elevates insulin levels through a dual mechanism: directly stimulating insulin secretion from β‐cells while alleviating the inhibitory effect of free fatty acids and non‐esterified fatty acids on insulin secretion. The resultant hyperinsulinemia is thought to activate the insulin/signal transducer and activator of transcription 5 (STAT5) signaling pathway in mammary cells, leading to phosphorylation of STAT5 transcription factors, which may enhance the efficiency of milk protein synthesis [75, 76]. Another study showed that SCFAs produced by *Vibrio butyricus* spp. in the intestinal tract can migrate to mammary tissues through blood circulation, where butyric acid indirectly regulates the epigenetic modification of lactoprotein genes (e.g., *CSN1S1*, *CSN2*) in mammary cells through the inhibition of histone deacetylases (HDACs) and activation of the peroxisome proliferator‐activated receptor gamma (PPARγ) nuclear receptor and transcriptional activity, thereby promoting milk protein synthesis in cows [81, 82]. Alterations of the gut microbial community can affect the efficiency of amino acid uptake and utilization by mammary cells, which in turn affect whey protein synthesis [83, 84]. Supplementation with leucine at 1.5 mM (equivalent to 10 μg per mammary gland daily) significantly upregulated whey protein (*β‐lactoglobulin*) and milk fat synthesis genes (*FASN*, *ACC*) in bovine mammary epithelial cells by activating the *mTOR/SREBP1* pathway [85]. Moreover, when the intestinal microecological balance is maintained, a stable immune environment promotes the secretion of high‐quality milk proteins by mammary cells [86]. For example, selenium, copper, and calcium supplementation enhance the abundance of *Lactobacillus* and *Bifidobacterium*, which produce SCFAs that attenuate mammary oxidative stress via Nrf2 signaling. This cascade restores immune homeostasis, promoting the synthesis of high‐quality milk proteins in dairy cows [87, 88].

#### Effect of gut‐mammary axis regulation on milk fat

Gut microbes can modulate the activity of fatty acid synthase in mammary cells, thereby altering the ratio of saturated to unsaturated fatty acids, and thus milk fat composition [89]. For example, in human models, lactic acid bacteria, through their expression of linoleic acid isomerase, converted dietary linoleic acid (LA) to cis‐9, trans‐11‐conjugated linoleic acid (c9, t11‐CLA), significantly elevating the CLA content of milk fat [90]. In a controlled feeding trial with lactating dairy cows, supplementation with fermented feed containing *Lactobacillus acidophilus* increased milk CLA content by 20%–30% [91]. CLA is an unsaturated fatty acid with various physiological functions in improving milk quality [92]. Some probiotics promote CLA production. For example, *Lactococcus lactis* generates CLA isomers by fermenting linoleic acid, increasing the percentage of c9,t11‐CLA in milk fat under optimized conditions [93]. *Bifidobacteria* also synthesize CLA through a similar pathway and regulate lipid metabolism in infants via mammary milk transmission [94, 95]. Triglycerides are the major form of milk fat, and intestinal microorganisms can alter triglyceride synthesis and secretion by affecting related signaling pathways, such as the peroxisome proliferator‐activated receptor (PPAR) signaling pathway, in mammary cells [96, 97]. Notably, the synergy between gut microbes and host metabolic pathways is not limited to gene expression regulation, but may also indirectly optimize the environment for milk fat synthesis by improving the metabolic status of the host. Yohe et al. [98] found that supplementation with Saccharomyces cerevisiae maintains the microecological balance of the digestive tract, improves the host's metabolic state, thereby increasing milk production and milk fat content in cows.

#### Influence of gut‐mammary axis modulation on other milk components

Lactose synthesis is relatively stable; however, the regulation of mammary cell metabolism by intestinal microorganisms can indirectly affect lactose synthesis [99]. For example, microbe‐regulated shifts in energy metabolism can affect the activity of key enzymes involved in lactose synthesis [100]. Some gut microbes can synthesize vitamins, such as vitamins K and B [101]. Consequently, these microbes can indirectly influence vitamin levels in milk by modulating their availability or synthesis in the gut of cows. For example, probiotics such as *Bifidobacteria* in the bovine gut synthesize vitamin B12, and changes in their abundance in the gut affect the level of vitamin B12 in milk [102, 103]. However, the quantitative contribution of intestinally synthesized B12 to the vitamin B12 content in milk remains poorly defined. Additionally, intestinal microorganisms can affect the mineral content of milk. Some gut microorganisms produce organic acids that lower the intestinal pH, promoting the dissolution and absorption of minerals such as calcium and phosphorus. These absorbed minerals are then transported via the bloodstream to mammary cells, ultimately influencing their concentration in milk [104].

#### Modulation of milk performance by gut‐mammary axis strategies

In livestock production, strategic adjustments to feeding management can effectively modulate the gut–mammary axis and gut health, thereby improving milk yield and quality [105, 106]. To compare the efficacy of different regulatory strategies, we summarized the key research findings in Table 1, which summarizes the effects of various regulatory strategies targeting the gut–mammary axis. These strategies improve milk quality through multiple pathways, including targeting the intestinal/mammary microenvironment (e.g., inulin and Lactobacillus salivarius), whereas other strategies enhance nutrient substrate utilization (e.g., LPL and SO + SSL) [107, 108]. Future development of precision nutrition strategies targeting the gut–mammary axis should prioritize SCFA‐based formulations to enhance STAT5‐mediated milk protein synthesis, engineered probiotic communities for species‐specific microbiota modulation, and real‐time monitoring of immune metabolism biomarkers to dynamically create interventions [109, 110, 111, 112]. These comprehensive approaches translate previous research into customized solutions for livestock production systems.

**Table 1:** Effects of gut–mammary axis regulation strategies on lactation performance.

Strategy	Mechanism	Study type	Experimental animals	Effect	Literature source
Concentrated pomegranate peel extract (CPE)	CPE synergizes milk quality and antioxidant capacity through the dual pathway of “direct antioxidant transfer + improved maternal metabolism”.	Controlled trial Add 0.36 kg dry matter/day of CPE (70 d)	Dairy goat (*n* = 58)	Improved milk quality and increased antioxidant capacity of milk in lactating ewes.	[107]
Astragalus membranaceus or Astragalus mongholicus (botany) (PTE)	Through the trinity mechanism of “antioxidant, anti‐inflammatory, and immunomodulation,” Astragalus improves the metabolic homeostasis of sows, reduces their own losses, and prioritizes the distribution of nutrients to the mammary glands, which ultimately improves the quality of milk.	Randomized trial Add 500 mg/kg of PTE (22 d)	Gestation sows (*n* = 10)	In a randomized trial of pregnant sows, Increased the immunoglobulin content of sow milk by 12% and reduced the level of inflammatory factor IL‐6 by 34%.	[108]
Phytobiocholine‐vitamin B dietary supplement	Choline‐vitamin B synergistically regulates liver metabolism, immunity, and mammary gland function to improve milk production and milk composition.	Controlled trial Add 10 g/day of vitamin B (20 d)	Lacaune sheep (*n* = 15)	Increased globulin concentration in milk and improved milk yield, feed conversion ratio, and milk SCC.	[109]
β‐Sitosterol	Inhibition of LPS‐induced inflammatory response in mammary epithelial cells and promotion of milk protein gene expression.	In vivo (mouse model) Add β‐sitosterol 1 μM + LPS 1 μg/mL (20 h)	Dairy cow (*n* = 3)	Expression of milk protein synthesis‐related genes (*CSN2*, *LALBA*) was up‐regulated 2.5‐fold.	[110]
hemolymphatic phospholipid (LPL)	LPL enhances fat digestion and absorption and increases glucose supply to sows by forming smaller fat particles and increasing the contact area for lipase, thus increasing the efficiency of fat hydrolysis.	Controlled trial Add 30 mg/kg of LPL (21 d)	Lactating sows (*n* = 16)	Increases the concentration of milk fat and lactose in milk.	[111]
Soybean oil (SO) + sodium stearoyl‐2‐lactate (SSL)	SSL synergistically improves the synthesis of milk fat, milk protein, and total solids by enhancing the efficiency of fat digestion and nutrient absorption in combination with the raw material supply of soybean oil.	Controlled trial Add 3% SO and 0.1% SSL (11 d)	Lactating sows (*n* = 30)	Increased milk fat, protein, and total solids concentrations in lactating sows on Day 11.	[112]

Abbreviations: CPE, concentrated pomegranate peel extract; LPL, hemolymphatic phospholipid; PTE, Astragalus membranaceus or Astragalus mongholicus (botany); SSL, sodium stearoyl‐2‐lactylate; SO, soybean oil.

### Impact of gut–mammary axis regulation on mammary gland health

Somatic cell counts in milk reflect the functional and immune status of the mammary glands, and gut microorganisms can regulate and improve somatic cell counts in dairy cow milk [113, 114]. Stabilization of the gut microbial community can regulate the expression of mammary epithelial cell‐associated proteins, enhance pathogen recognition and clearance, and modulate inflammatory signaling pathways, thereby controlling somatic cell count [115, 116, 117, 118]. For instance, in a mouse model of mastitis induced by *Staphylococcus aureus*, *Lactobacillus rhamnosus* significantly reduced somatic cell counts in milk by activating phagocytic activity in mammary macrophages and upregulating the expression of the anti‐inflammatory factor IL‐10 [119]. Butyric acid produced by *Clostridium butyricum* decreases somatic cell counts in cows with subclinical mastitis by inhibiting the TLR4/NF‐κB pathway [120, 121]. Moreover, microorganisms play a role in protein and fat metabolism to ensure cow nutrition and mammary gland health, and in regulating the secretion of stress hormones and protecting the intestinal barrier to mitigate the effects of stress, thereby reducing somatic cell counts [122, 123, 124]. Beneficial microorganisms enhance macrophage and neutrophil activity, regulate the secretion of anti‐inflammatory and pro‐inflammatory factors, and facilitate the normalization of somatic cell counts in milk [125, 126, 127]. For example, dietary supplementation with SCFAs reduces somatic cell counts and alleviate LPS‐induced inflammatory responses in dairy cows; however, effect sizes vary across studies [128]. The underlying mechanism involves inhibition of the NLRP3 signaling pathway, as detailed in Section 3.3.1. For instance, *Bifidobacteria* reduce somatic cell counts in milk by activating dendritic cells in the cow gut and promoting the differentiation of regulatory T cells (Tregs), thereby suppressing excessive inflammatory responses in the mammary glands [129]. *Bifidobacterium bifidum* inhibits excessive inflammatory responses in the mammary glands of dairy cows by activating intestinal dendritic cells, promoting differentiation of Tregs, and reducing somatic cell counts [130]. *Bacillus subtilis* reduces the risk of mammary infection by directly inhibiting the proliferation of *Streptococcus agalactiae* through the secretion of antimicrobial peptides, resulting in lower somatic cell counts in dairy cows [131, 132]. In addition, nutritional modulation can promote intestinal microecological balance and reduce mammary inflammatory responses via the gut‐mammary axis pathway. Notably, probiotic preparations (e.g., *Lactobacillus casei* Zhang and *Lactobacillus plantarum* P‐8) can promote cow health by maintaining intestinal microbiome homeostasis, increasing milk production, improving milk quality, and significantly reducing somatic cell counts in milk [133, 134].

Based on the delivery mechanism by which modulation of the gut‐mammary axis improves mammary health, the following application strategies have been popularized in animal husbandry (Table 2). Early warning models for mastitis can be constructed by monitoring dynamic changes in the intestinal microbiome. He et al. [14] found that intestinal microbiome disorders can induce mastitis in mice through the activation of the calcium‐ROS‐AMPK‐mTOR pathway, and that the elevated abundance of bacteria can serve as a high‐risk signal. In practice, dairy farms can screen for marker microorganisms in cow manure and combine machine learning algorithms to predict the probability of mastitis occurrence (By incorporating machine learning algorithms to predict the likelihood of mastitis, the reported prediction accuracy ranges from 94% to 98%.) and subsequently regulate the gut–mammary axis in a targeted manner to reduce the incidence rate and somatic cell count. Pilot studies of these technologies have been conducted in European and American ranches and may reduce economic losses by an average of approximately 12%–15% [142]. Based on the modulation of the gut‐mammary axis to improve mammary health, this study synthesized data from selected studies and summarized them in Table 2.

**Table 2:** Effects of gut–mammary axis regulation strategies on mammary health.

Specific protocol	Mechanism	Study type	Experimental animals	Effect	Literature source
Inulin	Inulin alleviates mammary gland inflammation in dairy cow by increasing the abundance of short‐chain fatty acid‐producing bacteria in the intestine, promoting the release of butyric, propionic, and acetic acids, and activating antimicrobial immunity in macrophages.	In vivo (mouse model) Add 300 g/day of inulin (56 d)	Dairy cow (*n* = 15)	Alleviated Staphylococcus aureus‐induced mastitis in dairy cow.	[135]
Sodium butyrate (NAB)	NAB reduces LPS and pro‐inflammatory cytokine concentrations and inhibits the NOD1/NF‐κB inflammatory pathway.	In vivo (goat) dose: not reported (168 d)	Goats (*n* = 6)	Dietary supplementation with sodium butyrate reduces mammary gland inflammation by modulating the NOD1/NF‐κB inflammatory pathway in SARA‐affected goat mammary glands.	[136]
*Lactobacillus salivarius* (PS2)	*Lactobacillus salivarius* PS2 reduces the incidence of mastitis by competitively inhibiting pathogenic bacterial colonization and modulating intestinal barrier function.	Randomized controlled trial Take approximately 1 × 10^9^ CFU of Lactobacillus salivarius PS2 orally once daily (Approximately 119 d)	Females (n = 328)	In a randomized controlled trial, Women in the probiotic group were 58% less likely to develop mastitis.	[137]
*Bacillus subtilis* (BS)C‐3102	*Bacillus subtilis* significantly reduces the risk of mastitis and improves milk quality through a multidimensional mechanism of competitive inhibition, immunomodulation, gut‐mammary axis interactions, antimicrobial secretion, antioxidant, and metabolic regulation.	In vivo (dairy cow) 20 g/d (3.0 × 10^9^ CFU) of BS) C‐3102 (11 mo)	Dairy cow (n = 10)	Significantly reduced the incidence of mastitis with mean SCC levels in milk.	[138]
Maslinicacid	Hawthorne acid down‐regulates mammary inflammation by inhibiting the activation of NLRP3 inflammatory vesicles, AKT/NF‐κB, and MAPK signaling pathways, and in addition, hawthorne acid protects the blood‐milk barrier by maintaining the expression of tight junction proteins.	In vivo (mouse model) Mslinicacid 100 mg/kg/d oral gavage (7 d)	Mice (*n* = 6)	Inhibits inflammatory reactions in the mammary gland, protects the blood‐milk barrier, and regulates the composition of the intestinal microbiome.	[139]
Astragalus polysaccharide (APS)	APS inhibits Staphylococcus aureus colonization, regulates inflammatory and oxidative responses, and protects the mammary barrier structure, thereby blocking infection, mitigating damage, and maintaining tissue function.	In vivo (mouse model) APS 200 mg/kg/d oral gavage (21 d)	Mice (*n* = 6)	APS reduces Staphylococcus aureus colonization in the mammary gland, modulates the inflammatory response, protects the blood‐milk barrier, and prevents oxidative stress, thereby alleviating Staphylococcus aureus‐induced mastitis.	[140]
Probio‐M9	Probio‐M9 enhances interactions between gut microbes, accompanied by an increase in the relative abundance of Ruminococcaceae and regulation of specific genes and carbohydrate‐active enzymes, thereby influencing host immunity.	In vivo (mouse model) dose: not reported (20 d)	Mice (*n* = 12)	Effectively alleviated mastitis, reduced inflammatory reactions, and improved response to antibiotic treatment.	[141]

Abbreviations: APS, Astragalus polysaccharide; CFU, colony‐forming unit; LPS, lipopolysaccharide; NAB, sodium butyrate; SCC, somatic cell count.

Collectively, interventions targeting the gut‐mammary axis enhance mammary health through three synergistic mechanisms: microbial metabolite signaling (e.g., SCFAs), immunomodulation (e.g., secretory IgA trafficking), and direct antimicrobial activity [135, 136]. Although probiotic efficacy in both ruminants and monogastrics is of interest, rumen‐specific challenges, such as maintaining probiotic viability in acidic environments, require tailored delivery systems [137]. Plant extracts have emerged as potent antibiotic alternatives by concurrently modulating multiple targets, underscoring the importance of integrating precision nutrition with scalable technologies for rapid translational impact [138]. Importantly, many mechanistic insights underlying these interventions originate from rodent or human studies [139, 140, 141]. Although conserved pathways have been confirmed in livestock, species‐specific differences in the gastrointestinal anatomy (rumen vs. monogastric), metabolic rates, and microbial ecology may alter the efficacy and optimal dosage of these interventions. Therefore, extrapolation from model organisms to production animals requires validation in the target species.

### Vertical transmission along the gut–mammary axis improves offspring performance and health

#### Effect of animal milk on the intestinal health of young animals

The colostrum is rich in immune‐related factors, and immunoglobulins are key components. Colostrum contains relatively high levels of immunoglobulin A (IgA), immunoglobulin G (IgG), and immunoglobulin M (IgM), which account for 70%–80% of the total protein in colostrum [143]. These immunoglobulins can be absorbed directly into the circulation by the intestines of the offspring, providing passive immunity during immune system development [144]. IgG can bind to antigens on the surface of pathogens, activate the complement system, and promote phagocytosis and clearance of pathogens by phagocytes, thus providing immediate immune protection to the offspring [145]. Donald et al. [146] found that milk secretory immunoglobulin A(sIgA) enhanced passive immunity in piglets by modulating the intestinal microbiota. In addition, lysozymes in milk can hydrolyze peptidoglycan found in the bacterial cell wall, destroying bacterial structure, killing the bacteria directly, reducing the chances of offsprings coming into contact with pathogens, and enhancing their immune defense ability [147, 148].

Milk plays an important role in promoting intestinal immune system development in offsprings. Lactoferrin is an important component of milk that promotes the proliferation and differentiation of intestinal epithelial cells and contributes to the maturation of the intestinal mucosal barrier [149]. A mature intestinal mucosal barrier effectively prevents pathogen invasion and reduces the risk of infection. Growth factors in animal milk, such as *Epidermal Growth Factor* (EGF) and *Insulin‐like Growth Factor* (IGF), stimulate the development of intestinal‐associated lymphoid tissues in offspring [150, 151, 152]. EGF promotes the expression of receptors on the surface of intestinal epithelial cells, strengthens intercellular junctions, and improves the intestinal barrier function [153, 154, 155]. In contrast, IGF can stimulate the proliferation and differentiation of intestinal lymphocytes and increase the activity and number of intestinal immune cells, thus enhancing intestinal immune function and promoting intestinal development [156]. The current challenges of artificial milk replacements in addressing intestinal developmental disorders and immune deficiencies in neonates necessitate functional biomimetic strategies based on bioactive components that are naturally present in maternal milk [157, 158, 159, 160, 161]. Table 3 summarizes five major categories of milk‐derived bioactive compounds and their evidence‐based functions in animal models, providing a theoretical basis for targeted interventions such as supplementation with β‐casein peptides and sialylated oligosaccharides.

**Table 3:** Milk bioactive compounds and their functional roles in neonatal development.

Category	Representative compounds	Major biological functions	Impact on offspring health	Literature sources
Vitamins & trace elements	Vitamin B12, Selenium, Zinc	Antioxidant defense Immune cell maturation	Reduced oxidative injury Enhanced immunoglobulin production	[152] [153]
Oligosaccharides	Sialylated human milk oligosaccharides (HMOs) Fucosyllactose	Protecting the intestinal barrier Pathogen adhesion blockade	Bifidobacterium dominance Lower necrotizing enterocolitis incidence	[154] [155]
Amino acids & peptides	β‐Casein peptides Epidermal Growth Factor (EGF)	Epithelial proliferation Antimicrobial action	Accelerated gut closure Pathogen colonization resistance	[156] [157]
Fatty acids	Conjugated linoleic acid (c9, t11‐CLA) Oleic acid	Metabolic programming Anti‐inflammatory effects	Optimized milk fat absorption Enhanced epithelial resilience	[158] [159]
Immunomodulatory proteins	Secretory IgA (sIgA) Lactoferrin Lysozyme	Pathogen neutralization Iron sequestration Bacterial lysis	Diarrhea reduction Immunit	[160] [161]

Abbreviations: CLA, conjugated linoleic acid; EGF, epidermal growth factor; HMOs, human milk oligosaccharides; sIgA, secretory immunoglobulin A.

#### Modulation of maternal gut–mammary axis improves offspring health

Offspring health can be improved by regulating the maternal gut–mammary axis (Figure 4). Animal milk secreted by the mammary glands contains specific microorganisms, such as lactic acid bacteria, which colonize the intestinal tract of young animals during lactation and shape the structure of their early intestinal microbiome [162]. Research has found that *bifidobacteria* in human breast milk can regulate the immune balance of Th17 cells in the intestines of young animals, thereby reducing the risk of inflammation [163]. This indicates that milk components from animals exert metabolic regulatory effects on the intestinal microbiota. Additionally, active immune substances in milk, such as lactoferrin and secretory immunoglobulin A (sIgA), inhibit pathogen colonization, maintain intestinal barrier function in young animals, and enhance nutrient absorption efficiency [164, 165, 166]. In the targeted optimization of milk components, adding lactoferrin or sIgA enhancers to the diet of lactating animals can increase the content of immune active substances in milk, significantly promoting intestinal barrier development and increasing the growth and weight gain of young animals [167]. Donald et al. [148] confirmed that animal milk sIgA influences immune development and prevents asthma by regulating the intestinal microbiota in mice. In terms of microbial vertical transfer interventions, maternal supplementation with *Lactobacillus* probiotics during pregnancy can colonize the intestines of young animals through the “maternal intestine mammary gland milk” pathway, shaping their early microbiota structure and reducing the risk of pathogen infections [168]. A meta‐analysis of randomized controlled trials and related studies revealed that maternal supplementation with probiotics can achieve strain‐specific vertical transmission to offspring through the gut–mammary axis, significantly increasing the abundance of specific probiotics (such as *Lactobacillus*) in mammary milk and potentially reducing pathogen colonization. Concurrently, the colonization of beneficial bacteria such as *Bifidobacterium* in the infant gut, and through the regulation of mammary milk components and immune factors, synergistically inhibits the proliferation of pathogenic bacteria, improves the immune system and gut microbiota of offspring, and reduces the incidence of infectious diseases, allergies, and necrotizing enterocolitis in offspring [169, 170, 171]. Wang et al. [172] found that feeding sows *Lactobacillus reuteri* regulates and accelerates the maturation of the early fecal microbiota composition of piglets, thereby improving their intestinal health. Additionally, adding lactoferrin or sIgA enhancers to the diet of sows can significantly increase the levels of immunologically active substances in animal milk, thereby promoting the development of the intestinal barrier in young animals and increasing their physical growth and weight gain [173, 174].

**Figure 4:**
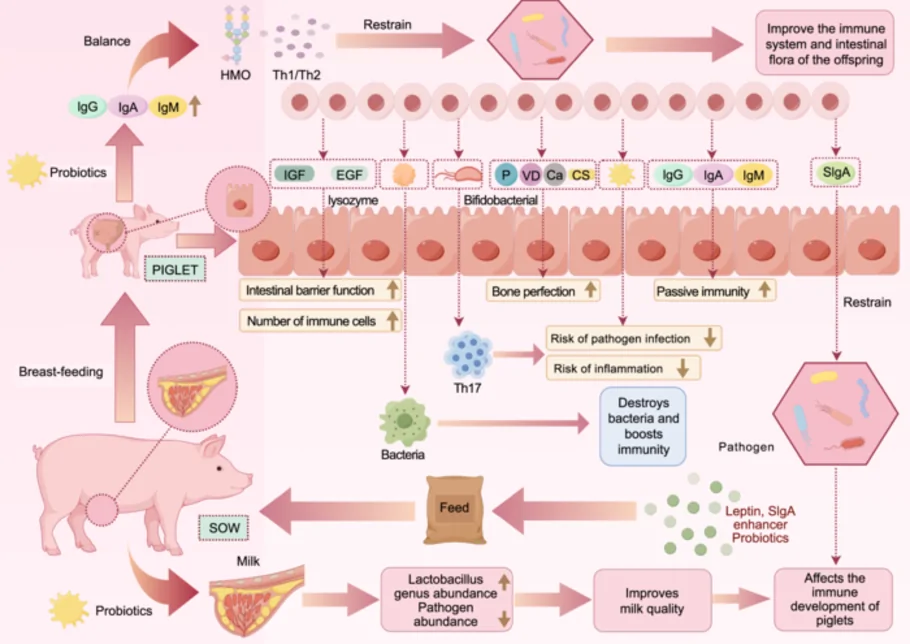
Vertical transmission via the gut–mammary axis improves offspring health and performance. Probiotic modulation of gut microbiota and immune homeostasis in piglets: Th1/Th2 balance, secretory IgA/SIgA dynamics, and cross‐organ effects on intestinal barrier integrity, bone mineralization, and mammary‐derived passive immunity, with translational implications for neonatal health and dairy production. Ca, calcium; CS, cesium; EGF, epidermal growth factor; HMO, human milk oligosaccharides; IGF, insulin‐like growth factor; LF, leptin; Th1/Th2, T helper 2 cell/T helper 2 cell; P, phosphorus; VD, vitamin D; TH17, T helper cell, 17.

The efficiency of maternal to offspring microbial transmission via the gut–mammary axis is not uniform but is influenced by several factors. Maternal diet plays a critical role: dietary fiber and prebiotics (e.g., inulin and fructooligosaccharides) enhance the abundance of beneficial bacteria, such as Bifidobacterium and Lactobacillus, in the maternal gut, thereby increasing their availability for translocation to the mammary glands [135, 170]. Lactation stage also modulates transmission, as colostrum typically contains higher microbial diversity and load than mature milk, suggesting a window of enhanced transfer in the early postnatal period. Additionally, maternal stress, antibiotic use, and parity may affect the gut microbiota composition and, consequently, the pool of vertically transmitted microbes.

In addition to immediate health benefits, the vertical transmission of specific maternal microbes has been linked to long‐term productive traits in the offspring. In piglets, early colonization with *Lactobacillus reuteri* via maternal supplementation reduced post‐weaning diarrhea, and improved the average daily gain and feed conversion ratio during the growing phase [172]. In dairy calves, the composition of the initial gut microbiota, shaped by colostrum‐derived microbes, correlates with subsequent weight gain and age at first estrus [162]. Moreover, retrospective studies suggest that the early life gut microbial profile may influence lifetime milk production efficiency, possibly through the programming of metabolic pathways and immune setpoints. These findings underscore the potential of targeting the maternal gut‐mammary axis as a long‐term strategy for the genetic and nutritional improvement of livestock productivity, complementing conventional breeding programs. Thus, offspring health can be managed by regulating the maternal gut–mammary axis.

### Species‐specific considerations in gut–mammary axis research

The gut–mammary axis has been investigated across multiple species, each serving distinct roles in the research continuum from mechanistic discovery to translational application. However, direct extrapolation of the findings between species requires careful consideration of anatomical, physiological, and microbiological differences (Figure 5).

**Figure 5:**
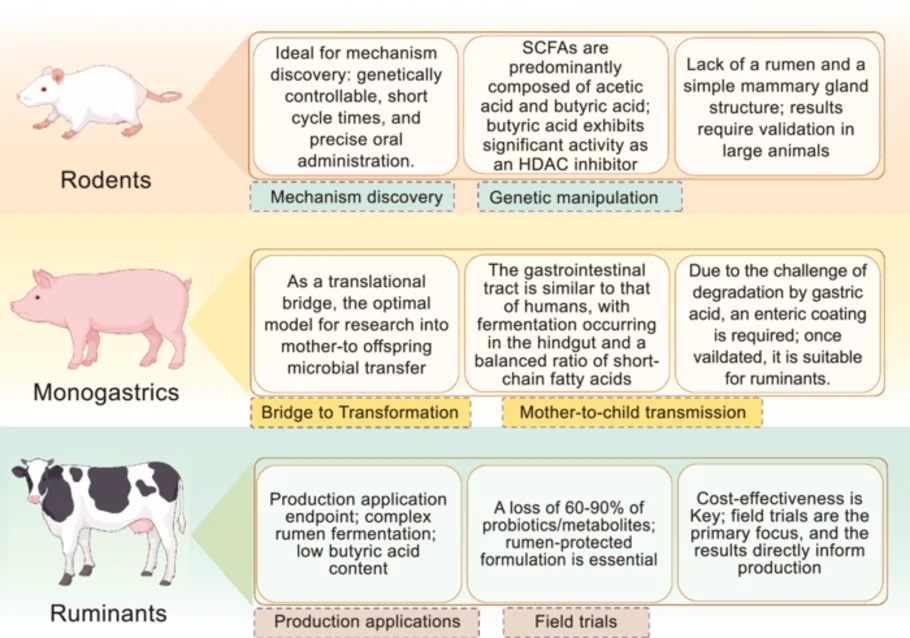
Species‐specific characteristics of the gut–mammary axis in rodents, pigs, and ruminants. Key species‐specific features of the gut–mammary axis across three animal categories are summarized. Rodents are characterized by short‐chain fatty acid (SCFA) profiles dominated by acetic and butyric acid (butyrate acts as a histone deacetylase inhibitor), lack a rumen, and require validation in larger animals. Pigs share a human‐like gastrointestinal tract with hindgut fermentation and a balanced SCFA ratio; gastric acid degradation necessitates enteric protection, and once validated, they serve as a translational bridge to ruminants. Ruminants exhibit low butyric acid, high probiotic/metabolite loss (60%–90%) due to rumen degradation, making rumen‐protected formulations essential; cost‐effectiveness is the primary focus, and results directly inform production practices.

Rodent models (mice and rats) are invaluable for elucidating fundamental mechanisms, including immune cell trafficking, epigenetic regulation, and microbial metabolite signaling. The advantages of using rodents include genetic tractability, precise experimental control, and short reproductive cycles. However, rodent models have inherent limitations; they lack a rumen ecosystem, have different mammary gland architectures, and do not fully mimic the metabolic demands of high‐producing livestock. Therefore, findings from rodent studies should be considered as a mechanistic proof of concept that requires validation in target livestock species. To bridge this gap, monogastric livestock serve as physiologically relevant intermediates.

Monogastric livestock (pigs) share a close gastrointestinal anatomy with humans and rodents, making them valuable for translational studies that aim to provide mechanistic insights from rodent to large animal applications. Pigs are particularly relevant for studying maternal offspring microbiome transmission and neonatal intestinal development, as their physiology mimics that of human infants more closely than that of rodents. However, the ultimate target for production‐oriented applications is ruminants, which pose the greatest challenge for intervention delivery.

Ruminants (dairy cows, goats, and sheep) are the primary targets for production‐oriented applications. The rumen introduces unique complexities, including extensive microbial fermentation, biohydrogenation of unsaturated fatty acids, and metabolic interactions that can either enhance or attenuate the effects of dietary interventions. For example, probiotic efficacy in ruminants is constrained by survival in an acidic ruminal environment, necessitating rumen‐protected formulations or post‐ruminal delivery strategies. Similarly, SCFA profiles differ substantially between ruminants (acetate and propionate dominance) and monogastrics (butyrate enrichment), which may influence downstream signaling via G protein‐coupled receptor 41/43 (GPR41/43) receptors. These species‐specific traits highlight the fact that no single model can fully recapitulate the gut–mammary axis of production animals. A tiered approach, starting with rodent mechanisms, moving to pig validation, and finally to ruminant applications, is essential for successful translation.

## 5 FUTURE PERSPECTIVES

### New discoveries in the regulatory mechanisms of the gut–mammary axis

With the recent rapid development of molecular biology, bioinformatics, and systems biology, scientists have gained a deeper understanding of the regulatory mechanisms underlying the gut–mammary axis. Epigenetic modifications, including DNA methylation, histone modifications, and non‐coding RNAs, have emerged as key mediators of the long‐term effects of gut microbiota on mammary gland function. Accumulating evidence indicates that traditional neural, endocrine, and immune regulatory pathways play an important role in the interaction between the gut and mammary axis, as do new regulatory factors, such as epigenetic modifications (e.g., DNA methylation and histone modifications) and gut microbiota metabolites [175, 176]. The oral administration of berberine increases the expression of miRNA‐145‐5p and miRNA‐34a‐5p in mouse mammary cells and reduces the formation of mammary glands in mouse mammary tumors [177]. In addition to miRNAs, long non‐coding RNAs (*lncRNAs*) have emerged as critical regulators of mammary gland development and function. The *H19 lncRNA*, for example, is an imprinted gene that controls mammary epithelial cell proliferation, apoptosis, and differentiation. Its expression is tightly regulated by DNA methylation at the *H19/IGF2 locus* and modulated by gut microbiota‐derived metabolites and inflammatory signals [178, 179]. *H19* dysregulation has been linked to aberrant mammary tissue remodeling and increased susceptibility to mastitis, suggesting that *lncRNAs* may serve as a mechanistic link between gut microbial ecology and mammary health. In addition, Ivanova et al [178]. demonstrated that gut microbiota‐derived metabolites and immune signals remotely regulate the DNA methylation of mammary genes in female mice. For instance, SCFAs such as butyrate can inhibit HDAC activity, leading to histone hyperacetylation and altered gene expression, whereas folate and other methyl donors from microbial synthesis influence DNA methyltransferase (DNMT) activity and DNA methylation patterns. Specifically, the methylation dynamics at the imprinted *IGF2R locus* form a bidirectional network that influences mammary development, lactation, and inflammatory responses. Hypermethylation of the *IGF2R* promoter has been associated with reduced expression and impaired mammary epithelial cell turnover during involution. *H19* encodes a long non‐coding RNA that regulates cell proliferation and apoptosis. Its methylation status is sensitive to gut microbial signals, and dysregulation may predispose mammary tissue to aberrant remodeling. Specifically, methylation dynamics at the imprinted *IGF2R locus* form a bidirectional network that influences mammary development, lactation, and inflammatory responses. For example, gut microbiota imbalance may interfere with the proliferation and apoptotic balance of mammary epithelial cells by inhibiting the demethylation of the *H19* gene (imprinted gene), thereby potentially increasing the risk of breast cancer in humans, ruminants, and monogastric animals, though such pathology is rare in livestock [180]. These findings indicate the complexity and diversity of gut–mammary axis regulation and provide new directions and ideas for future research.

### Research progress on intestine microbiota and mammary gland health

Recent research on the association between the gut microbiota and mammary health has revealed the multidimensional role of the gut–mammary axis in regulating the occurrence and development of mammary diseases through metabolic, immune, and epigenetic mechanisms. Mechanistic studies show that the human gut microbiota promotes the development of estrogen receptor‐positive mammary cancer through estrogen recycling mediated by β‐glucuronidase [180]. These cancer‐derived findings are used solely to illustrate conserved signaling mechanisms; the same pathways regulate lactation and mastitis in livestock. In contrast, metabolites such as SCFAs enhance mammary barrier function and alleviate inflammatory responses by inhibiting HDACs and activating GPR43 receptors [181]. Although estrogen‐dependent mammary pathology is not a major production issue, this mechanism underscores the importance of gut microbial metabolism in shaping the hormonal environment that influences mammary function and inflammation. Epigenetic evidence has shown that inflammatory signals associated with mastitis can reduce DNA methylation by regulating DNA methyltransferase (DNMT) activity and inducing DNA methylation reprogramming in the promoter region of the lactoprotein gene, thereby inhibiting lactation function in dairy cows [182, 183]. The human maternal gut microbiota influences the methylation patterns of genes related to mammary development in the offspring through mammary milk microbial metabolites, suggesting the potential for intergenerational regulation [184, 185]. In practical applications, strategies such as probiotic intervention (e.g., *Lactobacillus rhamnosus*) [186], dietary fiber supplementation [187], and microbiota transplantation [188] have demonstrated the potential to regulate the mammary immune microenvironment in dairy cows, pregnant mice, sows, and humans, thereby enhancing chemotherapy sensitivity. However, the species‐specific effects of the gut microbiota, spatiotemporal dynamics of host microbiota interactions, and standardized intervention protocols require in‐depth analysis through multi‐omics integration studies to provide new targets for the precise prevention and treatment of mammary diseases [189]. In addition, the gut microbiota can produce various biologically active metabolites, such as SCFAs and bile acids, which can influence the proliferation, differentiation, and apoptosis of mammary epithelial cells, thereby exerting a significant impact on mammary gland health [190]. Zhao et al. [191] found that bile acids produced by the gut microbiota of mice reduced *Staphylococcus aureus*‐induced mastitis. Future studies should elucidate the species‐specific mechanisms of gut microbiota‐derived metabolites in mammary pathogenesis, develop targeted delivery systems for microbial therapeutics, and validate clinical biomarkers such as fecal *Aeromonas* abundance coupled with serum sIgA to enable the precise prevention of mastitis across livestock species.

### Potential application of the gut–mammary axis in nutritional intervention and health management

The regulatory mechanisms of the gut–mammary axis provide new strategies and directions for nutritional intervention and health management. First, mammary gland development and milk performance in animals can be significantly improved by regulating intestinal health [192]. For example, the addition of probiotics and prebiotics, which are intestinal regulators, can improve the intestinal microenvironment and increase nutrient absorption efficiency [193]. Second, nutritional interventions, such as amino acids, fatty acids, and minerals, can meet the nutritional needs of mammary development and lactation [194]. Simultaneously, the adoption of precise feeding management measures can reduce stress responses and disease incidence in animals, thereby promoting mammary gland health [195]. In addition, by monitoring physiological indicators in the intestines and mammary glands and analyzing changes in related metabolic products, early warnings and precise interventions can be provided to monitor the health status of the animals [196]. Research has shown that *Stenotrophomonas maltophilia* can increase the permeability of the blood‐milk barrier by activating the calcium ROS‐AMPK‐mTOR autophagy pathway, thereby inducing mastitis in mice. This also demonstrates that targeted regulation of the gut‐mammary axis may be an effective method for treating mastitis [197]. Javier de Andrés et al. [198] found that *Lactobacillus salivarius* PS2 significantly reduced the incidence of mastitis in human females. These potential applications provide strong support for improving animal production performance and ensuring the health of both animals and humans. Extending this concept to ruminant production, recent longitudinal studies on dairy cows have identified rumen microbiota‐derived alanine as a key metabolite linking gut microbial ecology to host energy metabolism and lactation performance [199]. Specifically, alanine deficiency due to rumen dysbiosis was associated with ketosis and reduced milk protein content, whereas alanine supplementation alleviated ketogenesis in vitro, reinforcing the potential of targeting specific microbial metabolites as precise nutritional strategies for metabolic health management in livestock [200, 201].

Despite the promise of gut–mammary axis targeted interventions in controlled studies, commercial translation faces practical barriers. Cost effectiveness remains paramount, as production and delivery costs must be justified by on‐farm returns, especially in ruminants where rumen degradation necessitates expensive protected formulations. Scalability is hindered by the need for a sophisticated manufacturing infrastructure to maintain probiotic viability. Standardization is complicated by protocol variability and inconsistent active ingredient concentrations in plant extracts, necessitating industry‐wide consensus through meta‐analyses and multicenter trials. Regulatory approval processes are lengthy and costly and producer acceptance depends on clear returns on investment, ease of use, and compatibility with existing farm systems. Addressing these challenges requires collaborative efforts among researchers, feed manufacturers, veterinarians, and producers focusing on cost‐benefit analyses, scalable rumen‐protected formulations, standardized protocols, and commercial‐scale demonstration projects.

## 6 CONCLUSION

The gut‐mammary axis represents a functional bidirectional network that integrates intestinal and mammary physiology through circulating microbial metabolites, neuroendocrine signals, and immune cell trafficking. This review systematically presented evidence indicating that this axis operates through three interconnected layers. First, microbial metabolites modulate mammary epithelial cell function via HDAC inhibition, GPR41/43 activation, and STAT5/PPARγ signaling. Second, immune cell homing, particularly CCR10‐mediated trafficking of IgA+ plasma cells from gut‐associated lymphoid tissues to the mammary glands, establishes a direct link between maternal intestinal immunity and neonatal passive protection. Third, neuroendocrine mediators coordinate nutrient partitioning and metabolic adaptation to support lactation. The translational value of these mechanistic insights lies in their application in livestock production. Targeted nutritional interventions, including probiotic and prebiotic supplementation, SCFAs formulations, and plant‐derived bioactive compounds, have demonstrated efficacy in enhancing milk protein and fat synthesis, reducing somatic cell count, and mitigating mastitis risk in dairy cows, sows, and goats. Moreover, modulation of the maternal gut–mammary axis during gestation and lactation offers a powerful strategy for programming offspring gut microbiota and immune development, thereby improving neonatal health and reducing reliance on antibiotics.

Despite these advances, several critical challenges remain. First, species‐specific differences in the gastrointestinal anatomy, microbial ecology, and metabolic demands necessitate the development of tailored intervention strategies rather than a one‐size‐fits‐all approach. Second, most mechanistic evidence has been derived from rodent models, which, although invaluable for pathway discovery, do not fully recapitulate the physiological complexity of high‐producing livestock. Third, the cost‐effectiveness and scalability of precision nutrition interventions in commercial production systems remain largely unvalidated. Fourth, the bidirectional nature of the gut‐mammary axis, particularly how mammary‐derived factors affect intestinal function, remains underexplored.

We propose three directions for future research. First, multiomics integration (metagenomics, metabolomics, and epigenomics) across species should be leveraged to identify evolutionarily conserved regulatory nodes that can serve as universal targets for interventions. Second, next‐generation delivery systems, such as rumen‐protected formulations, encapsulation technologies, and maternal dietary programming, should be developed to overcome species‐specific barriers to probiotic and metabolite efficacy. Third, real‐time biomarker panels should be validated to provide early warning of mammary inflammation and monitoring of intervention responses in precision farming systems. By bridging the mechanistic understanding with species‐adapted nutritional strategies, the gut–mammary axis holds transformative potential for sustainable livestock production. Realizing this potential requires close collaboration between nutritionists, microbiologists, immunologists, and animal scientists to translate laboratory discoveries into cost‐effective and scalable solutions that enhance animal health, productivity, and welfare.

## AUTHOR CONTRIBUTIONS

Aolong Zhou, Cheng Guo, Yanliang Bi, Xiaofeng Xu, and Guijie Zhang conceived and designed the study. Aolong Zhou, Cheng Guo, and Pei Zhong developed the methodology. Aolong Zhou, Pei Zhong, and Jiawei Zhang performed the software analysis. Aolong Zhou, Cheng Guo, Jiawei Zhang, Junwei Cui, Qingyuan Yu, and Caijuan Yue conducted the formal analysis. Mahmoud M. Abdelsattar, Ahmad R. Musa, Mequanint Biks, Abdullahi A. Musa, Shahnaz Rahman, Abera Teshome Aleli, and Dina Karmallah performed the investigation. Naifeng Zhang, Tao Ma, Yan Tu, and Qiyu Diao provided resources. Cheng Guo, Xiyan Kou, Qingyuan Yu, Caijuan Yue, Qin Li, Dan Zhang, Zixuan Peng, Ao Ren, Ziqi Tian, Ziqi Liu, and Yaona Zhang curated the data. Aolong Zhou and Cheng Guo wrote the original draft. Lili Zhang, Xiaofeng Xu, Yanliang Bi, Guijie Zhang, Mahmoud M. Abdelsattar, and Dina Karmallah reviewed and edited the manuscript. Aolong Zhou and Cheng Guo prepared the visualizations. Yanliang Bi and Guijie Zhang supervised the study. Guijie Zhang, Xiaofeng Xu, and Yanliang Bi administered the project and acquired the funding. All authors have read the final manuscript and approved it for publication.

## CONFLICT OF INTEREST STATEMENT

The authors declare no conflicts of interest.

## ETHICS STATEMENT

No animals or humans were involved in this study.

## Data Availability

Data sharing is not applicable to this article as no datasets were generated or analyzed during the current study. No new data were created or analyzed in this study. Supplementary materials (graphical abstract, slides, videos, Chinese translated version, and updated materials) could be found in the online DOI or iMetaOmics http://www.imeta.science/imetaomics/.

## References

[1] Fried, Steven , Eve Wemelle , Patrice D. Cani , and Claude Knauf . 2021. “Interactions Between the Microbiota and Enteric Nervous System During Gut‐Brain Disorders.” Neuropharmacology 197: 108721. 10.1016/j.neuropharm.2021.108721 34274348

[2] Lan, Ying , Zhiyuan Ma , Lili Chang , Jing Peng , Mengqi Zhang , Qingyang Sun , Ruixue Qiao , et al. 2023. “Sea Buckthorn Polysaccharide Ameliorates High‐Fat Diet Induced Mice Neuroinflammation and Synaptic Dysfunction Via Regulating Gut Dysbiosis.” International Journal of Biological Macromolecules 236: 123797. 10.1016/j.ijbiomac.2023.123797 36828095

[3] Cruz, Clênio Silva , Mayra Fernanda Ricci , and Angélica Thomaz Vieira . 2021. “Gut Microbiota Modulation As a Potential Target for the Treatment of Lung Infections.” Frontiers in Pharmacology 12: 724033. 10.3389/fphar.2021.724033 PMC8453009 34557097

[4] Monteiro, Renato C. , and Laureline Berthelot . 2021. “Role of Gut‐Kidney Axis in Renal Diseases and IgA Nephropathy.” Current Opinion in Gastroenterology 37: 565–571. 10.1097/MOG.0000000000000789 34482323

[5] Boudry, Gaëlle , Elise Charton , Isabell Le Huerou‐Luron , Stéphanie Ferret‐Bernard , Sophie Le Gall , Sergine Even , and Sophie Blat . 2021. “The Relationship between Breast Milk Components and the Infant Gut Microbiota.” Frontiers in Nutrition 8: 629740. 10.3389/fnut.2021.629740 PMC8019723 33829032

[6] Oddy , Wendy H. 2026. “Breastmilk and immune function.” Annals of Nutrition and Metabolism. 10.1159/000550961. 41697895

[7] Selvamani, Shanmugaprakasham , Daniel Dailin , Vijai Gupta , Mohd Wahid , Ho Keat , Khairun Natasya , Roslinda Malek , et al. 2021. “An Insight into Probiotics Bio‐Route: Translocation from the Mother's Gut to the Mammary Gland.” Applied Sciences 11: 7247. 10.3390/app11167247

[8] Montoro‐Huguet, Miguel A. , Blanca Belloc , and Manuel Domínguez‐Cajal . 2021. “Small and Large Intestine (I): Malabsorption of Nutrients.” Nutrients 13: 1254. 10.3390/nu13041254. https://www.mdpi.com/2072-6643/13/4/1254 PMC8070135 33920345

[9] Pickard, Joseph M. , Melody Y. Zeng , Roberta Caruso , and Gabriel Núñez . 2017. “Gut Microbiota: Role in Pathogen Colonization, Immune Responses, and Inflammatory Disease.” Immunological Reviews 279: 70–89. 10.1111/imr.12567 PMC5657496 28856738

[10] Kuziel, Gavin A. , and Seth Rakoff‐Nahoum . 2022. “The Gut Microbiome.” Current Biology 32: R257–R264. 10.1016/j.cub.2022.02.023 35349808

[11] LeBlanc, Jean Guy , Florian Chain , Rebeca Martín , Luis G. Bermúdez‐Humarán , Stéphanie Courau , and Philippe Langella . 2017. “Beneficial Effects on Host Energy Metabolism of Short‐Chain Fatty Acids and Vitamins Produced By Commensal and Probiotic Bacteria.” Microbial Cell Factories 16: 79. 10.1186/s12934-017-0691-z PMC5423028 28482838

[12] Takiishi, Tatiana , Camila Ideli Morales Fenero , and Niels Olsen Saraiva Câmara . 2017. “Intestinal Barrier and Gut Microbiota: Shaping Our Immune Responses Throughout Life.” Tissue Barriers 5: e1373208. 10.1080/21688370.2017.1373208 PMC5788425 28956703

[13] Cani, Patrice D. , and Claude Knauf . 2016. “How Gut Microbes Talk to Organs: The Role of Endocrine and Nervous Routes.” Molecular Metabolism 5: 743–752. 10.1016/j.molmet.2016.05.011 PMC5004142 27617197

[14] Hu, Xiaoyu , Zhaoqi He , Caijun Zhao , Yuhong He , Min Qiu , Kaihe Xiang , Naisheng Zhang , and Yunhe Fu . 2024. “Gut/Rumen‐Mammary Gland Axis in Mastitis: Gut/Rumen Microbiota‐Mediated “Gastroenterogenic Mastitis.” Journal of Advanced Research 55: 159–171. 10.1016/j.jare.2023.02.009 PMC10770137 36822391

[15] Szajewska, Hania , Karen P. Scott , Tim de Meij , Sofia K. Forslund‐Startceva , Rob Knight , Omry Koren , Paul Little , et al. 2025. “Antibiotic‐Perturbed Microbiota and the Role of Probiotics.” Nature Reviews Gastroenterology & Hepatology 22: 155–172. 10.1038/s41575-024-01023-x 39663462

[16] Zhang, Jin , Qiqi Xie , Xingfa Huo , Zhilin Liu , Mengting Da , Mingxue Yuan , Yi Zhao , and Guoshuang Shen . 2022. “Impact of Intestinal Dysbiosis on Breast Cancer Metastasis and Progression.” Frontiers in Oncology 12: 1037831. 10.3389/fonc.2022.1037831 PMC9678367 36419880

[17] He, Zhaoqi , Caijun Zhao , Yuhong He , Zhuoyu Liu , Guyue Fan , Kun Zhu , Yiqi Wang , et al. 2023. “Enterogenic Stenotrophomonas Maltophilia Migrates to the Mammary Gland to Induce Mastitis by Activating the Calcium‐ROS‐AMPK‐mTOR‐Autophagy Pathway.” Journal of Animal Science and Biotechnology 14: 157. 10.1186/s40104-023-00952-y PMC10731779 38124149

[18] Yazdi, Mohammad Hossein , Mohammad Mehdi Soltan Dallal , Zuhair Mohammad Hassan , Marzieh Holakuyee , Solmaz Agha Amiri , Mohsen Abolhassani , and Mehdi Mahdavi . 2010. “Oral Administration of Lactobacillus Acidophilus Induces IL‐12 Production in Spleen Cell Culture of BALB/c Mice Bearing Transplanted Breast Tumour.” British Journal of Nutrition 104: 227–232. 10.1017/S0007114510000516 20193099

[19] Lakritz, Jessica R. , Theofilos Poutahidis , Tatiana Levkovich , Bernard J. Varian , Yassin M. Ibrahim , Antonis Chatzigiagkos , Sheyla Mirabal , Eric J. Alm , and Susan E. Erdman . 2014. “Beneficial Bacteria Stimulate Host Immune Cells to Counteract Dietary and Genetic Predisposition to Mammary Cancer in Mice.” International Journal of Cancer 135: 529–540. 10.1002/ijc.28702 PMC4131439 24382758

[20] Kassayova, Monika , Nikita Bobrov , Ladislav Strojny , Terezia Kiskova , Jaromir Mikes , Vlasta Demeckova , Peter Orendas , et al. 2014. “Preventive Effects of Probiotic Bacteria Lactobacillus Plantarum and Dietary Fiber in Chemically‐Induced Mammary Carcinogenesis.” Anticancer Research 34: 4969–4975. https://www.ncbi.nlm.nih.gov/pubmed/25202079 25202079

[21] Jiang, Hancai , Xiaoyu Mi , Zhenliang Zhu , Xin Li , Yong Zhang , and Jun Liu . 2025. “Mechanisms and Regulation of Mammary Gland Development in Dairy Animals.” Animal Advances 2: e025. 10.48130/animadv-0025-0022

[22] Rezaei, Reza , Zhenlong Wu , Yongqing Hou , Fuller W. Bazer , and Guoyao Wu . 2016. “Amino Acids and Mammary Gland Development: Nutritional Implications for Milk Production and Neonatal Growth.” Journal of Animal Science and Biotechnology 7: 20. 10.1186/s40104-016-0078-8 PMC4818943 27042295

[23] Hannan, Fadil M. , Taha Elajnaf , Laura N. Vandenberg , Stephen H. Kennedy , and Rajesh V. Thakker . 2023. “Hormonal Regulation of Mammary Gland Development and Lactation.” Nature Reviews Endocrinology 19: 46–61. 10.1038/s41574-022-00742-y 36192506

[24] Cabezuelo, M. Teresa , Rosa Zaragozá , Teresa Barber , and Juan R. Viña . 2020. “Role of Vitamin A in Mammary Gland Development and Lactation.” Nutrients 12: 80. 10.3390/nu12010080 PMC7019238 31892157

[25] Taylor, Vicky J . 2023. “Lactation from the Inside Out: Maternal Homeorhetic Gastrointestinal Adaptations Regulating Energy and Nutrient Flow into Milk Production.” Molecular and Cellular Endocrinology 559: 111797. 10.1016/j.mce.2022.111797 36243202

[26] Astbury, Stuart , Alison Mostyn , Michael E. Symonds , and Rhonda C. Bell . 2015. “Nutrient Availability, the Microbiome, and Intestinal Transport During Pregnancy.” Applied Physiology, Nutrition, and Metabolism 40: 1100–1106. 10.1139/apnm-2015-0117 26499849

[27] Teerapornpuntakit, Jarinthorn , Amornpan Klanchui , Nitsara Karoonuthaisiri , Kannikar Wongdee , and Narattaphol Charoenphandhu . 2014. “Expression of Transcripts Related to Intestinal Ion and Nutrient Absorption in Pregnant and Lactating Rats as Determined by Custom‐Designed cDNA Microarray.” Molecular and Cellular Biochemistry 391: 103–116. 10.1007/s11010-014-1992-8 24519337

[28] Ma, Xin , Yan Li , Chengyan Chen , Yanmin Shen , Hua Wang , and Tangliang Li . 2023. “Spatial Expression of the Nonsense‐Mediated mRNA Decay Factors UPF3A and UPF3B Among Mouse Tissues.” Journal of Zhejiang University‐Science B 24: 1062–1068. 10.1631/jzus.B2300126 PMC10646394 37961809

[29] Ye, Zi , Yu Xu , Mengna Zhang , and Cheguo Cai . 2024. “Sympathetic Nerve Signals: Orchestrators of Mammary Development and Stem Cell Vitality.” Journal of Molecular Cell Biology 16: mjae020. 10.1093/jmcb/mjae020 PMC11520406 38740522

[30] He, Yuhong , Yue Zhang , Lihua Zhao , Zeming Zhou , Nier Su , Can Zhang , Keyi Wang , et al. 2025. “Vagus Nerve Stimulation Alleviates S. Aureus‐Induced Mastitis by Regulating Gut Microbiota S24‐7‐PPARγ and NF‐ΚB/NLRP3 Signaling in Mice.” Journal of Neuroinflammation 23: 32. 10.1186/s12974-025-03654-6 PMC12838515 41430282

[31] Ni, Yifan , Qiangqiang Chen , Jianfeng Cai , Lixia Xiao , and Jinzhi Zhang . 2021. “Three Lactation‐Related Hormones: Regulation of Hypothalamus‐Pituitary Axis and Function on Lactation.” Molecular and Cellular Endocrinology 520: 111084. 10.1016/j.mce.2020.111084 33232781

[32] Dobolyi, Arpád , Szilvia Oláh , Dávid Keller , Rashmi Kumari , Emese A. Fazekas , Vivien Csikós , Éva Renner , and Melinda Cservenák . 2020. “Secretion and Function of Pituitary Prolactin in Evolutionary Perspective.” Frontiers in Neuroscience 14: 621. 10.3389/fnins.2020.00621 PMC7308720 32612510

[33] Yun, Chuyu , Sen Yan , Baoying Liao , Yong Ding , Xinyu Qi , Min Zhao , Kai Wang , et al. 2024. “The Microbial Metabolite Agmatine Acts as an FXR Agonist to Promote Polycystic Ovary Syndrome in Female Mice.” Nature Metabolism 6: 947–962. 10.1038/s42255-024-01041-8 38769396

[34] Spiljar, Martina , Doron Merkler , and Mirko Trajkovski . 2017. “The Immune System Bridges the Gut Microbiota with Systemic Energy Homeostasis: Focus on Tlrs, Mucosal Barrier, and Scfas.” Front Immunol 8: 1353. 10.3389/fimmu.2017.01353 PMC5670327 29163467

[35] Macfabe, Derrick . 2013. “Autism: Metabolism, Mitochondria, and the Microbiome.” Global Advances in Health and Medicine 2: 52–66. 10.7453/gahmj.2013.089 PMC3865378 24416709

[36] Huang, Gang , and Liang Chen . 2021. “Biological Activity of Biomimetic Dermal Papilla Spheres Prepared By Culture of Dermal Papilla Cells of Mice Based on Hanging Drops of Gelatin Methacrylate and Its Hair‐Inducing Function in Nude Mice.” Chinese Journal of Burns 37: 770–780. 10.3760/cma.j.cn501120-20210330-00109 PMC11917309 34404159

[37] Natale, G. , L. Ryskalin , C. L. Busceti , F. Biagioni , and F. Fornai . 2017. “The Nature of Catecholamine‐Containing Neurons in the Enteric Nervous System in Relationship with Organogenesis, Normal Human Anatomy and Neurodegeneration.” Archives Italiennes De Biologie 155: 118–130. 10.12871/00039829201733 29220864

[38] Long, Xiaoyang , Jing Ye , Di Zhao , and Sheng‐Jia Zhang . 2015. “Magnetogenetics: Remote Non‐Invasive Magnetic Activation of Neuronal Activity With a Magnetoreceptor.” Science Bulletin 60: 2107–2119. 10.1007/s11434-015-0902-0 PMC4692962 26740890

[39] Donowitz, Mark , Jerrold R. Turner , Alan S. Verkman , and Nicholas Constantine Zachos . 2020. “Current and Potential Future Applications of Human Stem Cell Models in Drug Development.” Journal of Clinical Investigation 130: 3342–3344. 10.1172/JCI138645 PMC7324189 32452833

[40] Farmer, C . 2013. “Suckling Effects in Sows: Importance for Mammary Development and Productivity.” Animal 7: 1964–1968. 10.1017/S1751731113001201 23842122

[41] Jiang, Hanyun , Sophie Gallier , Lifang Feng , Jianzhong Han , and Weilin Liu . 2022. “Development of the Digestive System in Early Infancy and Nutritional Management of Digestive Problems in Breastfed and Formula‐Fed Infants.” Food & Function 13: 1062–1077. 10.1039/d1fo03223b 35073567

[42] Sandoval, Darleen , and Stephanie R. Sisley . 2015. “Brain GLP‐1 and Insulin Sensitivity.” Molecular and Cellular Endocrinology 418: 27–32. 10.1016/j.mce.2015.02.017 PMC4547906 25724479

[43] Tong, Guoxiang , Tianhao Peng , Ya Chen , Lijuan Sha , Huikang Dai , Yidong Xiang , Zhiqi Zou , Heli He , and Sha Wang . 2022. “Effects of GLP‐1 Receptor Agonists on Biological Behavior of Colorectal Cancer Cells by Regulating PI3K/AKT/mTOR Signaling Pathway.” Frontiers in Pharmacology 13: 901559. 10.3389/fphar.2022.901559 PMC9399678 36034798

[44] Ligumsky, Hagai , Ido Wolf , Shira Israeli , Michal Haimsohn , Sarah Ferber , Avraham Karasik , Bella Kaufman , and Tami Rubinek . 2012. “The Peptide‐Hormone Glucagon‐Like Peptide‐1 Activates cAMP and Inhibits Growth of Breast Cancer Cells.” Breast Cancer Research and Treatment 132: 449–461. 10.1007/s10549-011-1585-0 21638053

[45] Gribble, Fiona M. , and Frank Reimann . 2019. “Function and Mechanisms of Enteroendocrine Cells and Gut Hormones in Metabolism.” Nature Reviews Endocrinology 15: 226–237. 10.1038/s41574-019-0168-8 30760847

[46] Meloni, A. R. , M. B. DeYoung , C. Lowe , and D. G. Parkes . 2013. “GLP‐1 Receptor Activated Insulin Secretion from Pancreatic Beta‐Cells: Mechanism and Glucose Dependence.” Diabetes Obesity & Metabolism 15: 15–27. 10.1111/j.1463-1326.2012.01663.x PMC3556522 22776039

[47] Sloth, Birgitte , Jens Juul Holst , Anne Flint , Nikolaj Ture Gregersen , and Arne Astrup . 2007. “Effects of pyy1‐36 and pyy3‐36 on Appetite, Energy Intake, Energy Expenditure, Glucose and Fat Metabolism in Obese and Lean Subjects.” American Journal of Physiology‐Endocrinology and Metabolism 292: E1062–E1068. 10.1152/ajpendo.00450.2006 17148749

[48] Sánchez, Manuel Lisardo , Francisco D. Rodríguez , and Rafael Coveñas . 2023. “Neuropeptide Y Peptide Family and Cancer: Antitumor Therapeutic Strategies.” International Journal of Molecular Sciences 24: 9962. 10.3390/ijms24129962 PMC10297964 37373115

[49] Persaud, Shanta J. , and Gavin A. Bewick . 2014. “Peptide YY: More Than Just an Appetite Regulator.” Diabetologia 57: 1762–1769. 10.1007/s00125-014-3292-y 24917132

[50] Sinkiewicz‐Darol, Elena , Iwona Adamczyk , Katarzyna Łubiech , Gabriela Pilarska , and Magdalena Twarużek . 2022. “Leptin in Human Milk—One of the Key Regulators of Nutritional Programming.” Molecules 27: 3581. 10.3390/molecules27113581 PMC9182392 35684517

[51] Le Dréan, Gwenola , and Jean‐Pierre Segain . 2014. “Connecting Metabolism to Intestinal Barrier Function: The Role of Leptin.” Tissue Barriers 2: e970940. 10.4161/21688362.2014.970940 PMC4292046 25610758

[52] Yarandi, Shadi S. , Gautam Hebbar , Cary G. Sauer , Conrad R. Cole , and Thomas R. Ziegler . 2011. “Diverse Roles of Leptin in the Gastrointestinal Tract: Modulation of Motility, Absorption, Growth, and Inflammation.” Nutrition 27: 269–275. 10.1016/j.nut.2010.07.004 PMC3066025 20947298

[53] Rajala, Michael W. , Christa M. Patterson , Judith S. Opp , Susan K. Foltin , Vincent B. Young , and Martin G. Myers . 2014. “Leptin Acts Independently of Food Intake to Modulate Gut Microbial Composition in Male Mice.” Endocrinology 155: 748–757. 10.1210/en.2013-1085 PMC3929727 24424041

[54] Dena‐Beltrán, José Luis , Ana Luisa Ocampo‐Ruiz , Dina I. Vázquez‐Carrillo , Ericka A. de los Ríos , Xarubet Ruiz‐Herrera , Magali Valle‐Pacheco , Sairi I. Robles‐Torres , et al. 2025. “Prolactin Maintains the Neonatal Phenotype of Enterocytes During Lactation in Newborn Mice.” American Journal of Physiology‐Cell Physiology 329: C17–C30. 10.1152/ajpcell.00981.2024 40353577

[55] Ding, Guoao , Xuezhi Yang , Ying Li , Ying Wang , Yujie Du , Meng Wang , Ruxin Ye , et al. 2025. “Gut Microbiota Regulates Gut Homeostasis, Mucosal Immunity and Influences Immune‐Related Diseases.” Molecular and Cellular Biochemistry 480: 1969–1981. 10.1007/s11010-024-05077-y 39060829

[56] Gou, Hongzhong , YuLin Zhang , LongFei Ren , Zhen‐Jiao Li , and Lei Zhang . 2022. “How Do Intestinal Probiotics Restore the Intestinal Barrier?” Frontiers in Microbiology 13: 929346. 10.3389/fmicb.2022.929346 PMC9330398 35910620

[57] Fischer‐Tlustos, A. J. , A. Lopez , K. S. Hare , K. M. Wood , and M. A. Steele . 2021. “Effects of Colostrum Management on Transfer of Passive Immunity and the Potential Role of Colostral Bioactive Components on Neonatal Calf Development and Metabolism.” Canadian Journal of Animal Science 101: 405–426. 10.1139/cjas-2020-0149

[58] Liang, Chunlan , Xile Mu , Qinglan Bao , Pengsigerexi Borzigin , Hongyan Sheng , Xiaomei Han , Yingsong Chen , and Tegexibaiyin Wang . 2024. “Exploring the Inhibitory Impact of Mongolian Medicinal He‐Zi‐3 Soup on Mammary Gland Hyperplasia in Rats Induced By Estrogen and Progestogen.” Journal of Ethnopharmacology 329: 117854. 10.1016/j.jep.2024.117854 38583733

[59] Reboldi, Andrea , and Jason G. Cyster . 2016. “Peyer's Patches: Organizing B‐Cell Responses at the Intestinal Frontier.” Immunological Reviews 271: 230–245. 10.1111/imr.12400 PMC4835804 27088918

[60] Reboldi, Andrea , and Jason G. Cyster . 2017. “Mucosal Chemokines.” Journal of Interferon & Cytokine Research 37: 62–70. 10.1089/jir.2016.0076 PMC5314990 28207301

[61] Morteau, Olivier , Craig Gerard , Bao Lu , Sorina Ghiran , Miriam Rits , Yuko Fujiwara , Yuetching Law , et al. 2008. “An Indispensable Role for the Chemokine Receptor ccr10 in Iga Antibody‐Secreting Cell Accumulation.” Journal of Immunology 181: 6309–6315. 10.4049/jimmunol.181.9.6309 PMC2748758 18941222

[62] Donald, Katherine , Charisse Petersen , Stuart E. Turvey , B. Brett Finlay , and Meghan B. Azad . 2022. “Secretory IgA: Linking Microbes, Maternal Health, and Infant Health through Human Milk.” Cell Host & Microbe 30: 650–659. 10.1016/j.chom.2022.02.005 35550668

[63] Barbara, Giovanni , Maria Raffaella Barbaro , Daniele Fuschi , Marta Palombo , Francesca Falangone , Cesare Cremon , Giovanni Marasco , and Vincenzo Stanghellini . 2021. “Inflammatory and Microbiota‐Related Regulation of the Intestinal Epithelial Barrier.” Frontiers in Nutrition 8: 718356. 10.3389/fnut.2021.718356 PMC8475765 34589512

[64] Nishizawa, Kazuhisa . 2016. “Low‐Grade Endotoxemia, Diet, and Gut Microbiota – An Emphasis on the Early Events Leading to Dysfunction of the Intestinal Epithelial Barrier.” Biomedical Research and Clinical Practice 1: 46–57. 10.15761/brcp.1000110

[65] Wang, Zhiwei , Zheng Ma , Zhichen Tian , Haoran Jia , Lei Zhang , Yongjiang Mao , Zhangping Yang , Xu Liu , and Mingxun Li . 2025. “Microbial Dysbiosis in the Gut‐Mammary Axis As a Mechanism for Mastitis in Dairy Cows.” International Journal of Dairy Technology 78: e13150. 10.1111/1471-0307.13150

[66] Yoo, Ji , Maureen Groer , Samia Dutra , Anujit Sarkar , and Daniel McSkimming . 2020. “Gut Microbiota and Immune System Interactions.” Microorganisms 8: 1587. 10.3390/microorganisms8101587 PMC7602490 33076307

[67] La Fata, Giorgio , Peter Weber , and M. Hasan Mohajeri . 2018. “Probiotics and the Gut Immune System: Indirect Regulation.” Probiotics and Antimicrobial Proteins 10: 11–21. 10.1007/s12602-017-9322-6 PMC5801397 28861741

[68] Cheong, Kit‐Leong , Jiajing Li , Min Wang , Dongju Wang , and Saiyi Zhong . 2026. “Cross‐Kingdom Signals: Microbial, Metabolites, and Immune Cells on the Gut‐Breast Axis.” Critical Reviews in Food Science and Nutrition 2: 1–18. 10.1080/10408398.2026.2681931. 42228631

[69] Wang, Chengqin , Manqin Zeng , Yufen An , Wenhong Wang , Xianning Dong , Guangming Fu , and Zhimin Wei . 2019. “Clinicopathological Analysis of Breast Primary Mucinous Cyst Adenocarcinoma.” Chinese Journal of Pathology 48: 242–244. 10.3760/cma.j.issn.0529-5807.2019.03.015 30831654

[70] Biswas, Swarajit Kumar , Saswati Banerjee , Ginger Wendolyn Baker , Chieh‐Yin Kuo , and Indrajit Chowdhury . 2022. “The Mammary Gland: Basic Structure and Molecular Signaling During Development.” International Journal of Molecular Sciences 23: 3883. 10.3390/ijms23073883 PMC8998991 35409243

[71] Zigo, František , Milan Vasil' , Silvia Ondrašovičová , Jana Výrostková , Jolanta Bujok , and Ewa Pecka‐Kielb . 2021. “Maintaining Optimal Mammary Gland Health and Prevention of Mastitis.” Frontiers in Veterinary Science 8: 607311. 10.3389/fvets.2021.607311 PMC7927899 33681324

[72] Zhang, Wei , Bello Babatunde Kazeem , Haitao Yang , Gang Liu , Guanglu Wang , Zhixing Li , Tao Guo , Panpan Zhao , and Jingquan Dong . 2021. “Aeromonas Sobria Regulates Proinflammatory Immune Response in Mouse Macrophages Via Activating the MAPK, AKT, and NF‐κB Pathways.” Journal of Zhejiang University‐Science B 22: 782–790. 10.1631/jzus.B2100456 PMC8435340 34514758

[73] Cao, Zhaohui , Di Huang , Cifei Tang , Min Zeng , and Xiaobo Hu . 2022. “PFKL, a Novel Regulatory Node for NOX2‐Dependent Oxidative Burst and NETosis.” Journal of Zhejiang University‐Science B 23: 607–612. 10.1631/jzus.B2101029 PMC9264108 35794690

[74] Finsterer, Josef . 2020. “Secondary Manifestations of Mitochondrial Disorders.” Journal of Zhejiang University‐Science B 21: 590–592. 10.1631/jzus.B2000010 PMC7383320 32633113

[75] Chen, Zhujun , Xinyi Zhang , Jun Teng , Li Jiang , and Qin Zhang . 2025. “HSF1 and CPSF1 Affect Milk Fat and Protein Synthesis by Regulating the AKT/mTOR Signaling Pathway.” Journal of Animal Science 103: skaf009. 10.1093/jas/skaf009 PMC11897896 39932399

[76] Pawłowski, Karol , José A. A. Pires , Yannick Faulconnier , Christophe Chambon , Pierre Germon , Céline Boby , and Christine Leroux . 2019. “Mammary Gland Transcriptome and Proteome Modifications by Nutrient Restriction in Early Lactation Holstein Cows Challenged with Intra‐Mammary Lipopolysaccharide.” International Journal of Molecular Sciences 20: 1156. 10.3390/ijms20051156 PMC6429198 30845783

[77] Zhang, Yongyun , Xinyang Fan , Lihua Qiu , Wei Zhu , Lige Huang , and Yongwang Miao . 2021. “Liver X Receptor α Promotes Milk Fat Synthesis in Buffalo Mammary Epithelial Cells by Regulating the Expression of FASN.” Journal of Dairy Science 104: 12980–12993. 10.3168/jds.2021-20596 34593221

[78] Wang, Yue , Xuemei Nan , Yiguang Zhao , Linshu Jiang , Hui Wang , Dengke Hua , Fan Zhang , et al. 2021. “Dietary Supplementation with Inulin Improves Lactation Performance and Serum Lipids by Regulating the Rumen Microbiome and Metabolome in Dairy Cows.” Animal Nutrition 7: 1189–1204. 10.1016/j.aninu.2021.09.007 PMC8556608 34754961

[79] Allen, M. S . 2020. “Review: Control of Feed Intake By Hepatic Oxidation in Ruminant Animals: Integration of Homeostasis and Homeorhesis.” Animal 14: s55–s64. 10.1017/S1751731119003215 32024573

[80] Sivri, Dilek , and Yasemin Akdevelioğlu . 2025. “Effect of Fatty Acids on Glucose Metabolism and Type 2 Diabetes.” Nutrition Reviews 83: 897–907. 10.1093/nutrit/nuae165 PMC11986341 39530757

[81] Guo, Wenjin , Juxiong Liu , Jingxuan Sun , Qian Gong , He Ma , Xingchi Kan , Yu Cao , Jianfa Wang , and Shoupeng Fu . 2020. “Butyrate Alleviates Oxidative Stress By Regulating NRF2 Nuclear Accumulation and H3K9/14 Acetylation Via GPR109A in Bovine Mammary Epithelial Cells and Mammary Glands.” Free Radical Biology and Medicine 152: 728–742. 10.1016/j.freeradbiomed.2020.01.016 31972340

[82] Chen, Jingbo , Yongjiang Wu , Yawang Sun , Xianwen Dong , Zili Wang , Zhu Zhang , Yanli Xiao , and Guozhong Dong . 2019. “Bacterial Endotoxin Decreased Histone H3 Acetylation of Bovine Mammary Epithelial Cells and the Adverse Effect Was Suppressed by Sodium Butyrate.” BMC Veterinary Research 15: 267. 10.1186/s12917-019-2007-5 PMC6664593 31357995

[83] Dai, Z. , Z. Wu , S. Hang , W. Zhu , and G. Wu . 2015. “Amino Acid Metabolism in Intestinal Bacteria and Its Potential Implications for Mammalian Reproduction.” Molecular Human Reproduction 21: 389–409. 10.1093/molehr/gav003 25609213

[84] Wang, Feiran , Haitao Shi , Shuxiang Wang , Yajing Wang , Zhijun Cao , and Shengli Li . 2019. “Amino Acid Metabolism in Dairy Cows and Their Regulation in Milk Synthesis.” Current Drug Metabolism 20: 36–45. 10.2174/1389200219666180611084014 29886827

[85] Huang, Jiangtao , Jintao Li , Yong Ning , Yalun Ren , Yuexin Shao , Huawen Zhang , Xueyang Zong , and Huaiping Shi . 2023. “Enhancement of PPARα‐Inhibited Leucine Metabolism‐Stimulated β‐Casein Synthesis and Fatty Acid Synthesis in Primary Bovine Mammary Epithelial Cells.” Journal of Agricultural and Food Chemistry 71: 16184–16193. 10.1021/acs.jafc.3c00712 37853551

[86] Zhang, Chao , Houyu Chi , Xueting Han , Jie Zheng , Sijia Yang , Ai Li Li , and Wei Yu . 2023. “Advances in the Protection of Intestinal Mucosal Barrier Function by Milk‐Derived miRNAs.” Food & Function 14: 3909–3928. 10.1039/d3fo00137g 37016752

[87] Khan, Muhammad Zahoor , Bingjian Huang , Xiyan Kou , Yinghui Chen , Huili Liang , Qudrat Ullah , Ibrar Muhammad Khan , et al. 2023. “Enhancing Bovine Immune, Antioxidant and Anti‐Inflammatory Responses With Vitamins, Rumen‐Protected Amino Acids, and Trace Minerals to Prevent Periparturient Mastitis.” Frontiers in Immunology 14: 1290044. 10.3389/fimmu.2023.1290044 PMC10800369 38259482

[88] Xiao, Jianxin , Muhammad Zahoor Khan , Yulin Ma , Gibson Maswayi Alugongo , Jiaying Ma , Tianyu Chen , Adnan Khan , and Zhijun Cao . 2021. “The Antioxidant Properties of Selenium and Vitamin E; Their Role in Periparturient Dairy Cattle Health Regulation.” Antioxidants 10: 1555. 10.3390/antiox10101555 PMC8532922 34679690

[89] Klinsawat, Worata , Pichahpuk Uthaipaisanwong , Piroon Jenjaroenpun , Supaphen Sripiboon , Thidathip Wongsurawat , and Kanthida Kusonmano . 2023. “Microbiome Variations Among Age Classes and Diets of Captive Asian Elephants (Elephas maximus) in Thailand Using Full‐Length 16S rRNA Nanopore Sequencing.” Scientific Reports 13: 17685. 10.1038/s41598-023-44981-z PMC10582034 37848699

[90] González, Adriana , Asier Fullaondo , Javier Rodríguez , Cristina Tirnauca , Iñaki Odriozola , and Adrian Odriozola . 2025. “Conjugated Linoleic Acid Metabolite Impact in Colorectal Cancer: A Potential Microbiome‐Based Precision Nutrition Approach.” Nutrition Reviews 83: e602–e614. 10.1093/nutrit/nuae046 PMC11723137 38728013

[91] Meng, Li , Shuang Li , Gefei Liu , Xuejing Fan , Yali Qiao , Ao Zhang , Yanan Lin , et al. 2021. “The Nutrient Requirements of Lactobacillus Acidophilus LA‐5 and Their Application to Fermented Milk.” Journal of Dairy Science 104: 138–150. 10.3168/jds.2020-18953 33131816

[92] Wang, Kun , Zimeng Xin , Zhi Chen , Huanan Li , Diming Wang , and Yuan Yuan . 2023. “Progress of Conjugated Linoleic Acid on Milk Fat Metabolism in Ruminants and Humans.” Animals 13: 3429. 10.3390/ani13213429 PMC10647460 37958184

[93] Chen, Cunshe , Fang Tong , Ruohao Sun , Ying Zhang , Zhihua Pang , and Xinqi Liu . 2024. “Screening and Identification of High‐Yielding Strains of Conjugated Linoleic Acid and Optimization of Conditions for the Conversion of CLA.” Foods 13: 1830. 10.3390/foods13121830 PMC11203061 38928772

[94] Mei, Yongchao , Haiqin Chen , Bo Yang , Jianxin Zhao , Hao Zhang , and Wei Chen . 2021. “Linoleic Acid Triggered a Metabolomic Stress Condition in Three Species of Bifidobacteria Characterized By Different Conjugated Linoleic Acid‐Producing Abilities.” Journal of Agricultural and Food Chemistry 69: 11311–11321. 10.1021/acs.jafc.1c03752 34523917

[95] Lordan, Cathy , Aoife K. Roche , Dianne Delsing , Arjen Nauta , Andre Groeneveld , John MacSharry , Paul D. Cotter , and Douwe van Sinderen . 2024. “Linking Human Milk Oligosaccharide Metabolism and Early Life Gut Microbiota: Bifidobacteria and Beyond.” Microbiology and Molecular Biology Reviews 88: e0009423. 10.1128/mmbr.00094-23 PMC10966949 38206006

[96] Qiu, Yuan‐Ye , Jing Zhang , Fan‐Yi Zeng , and Yi Zhun Zhu . 2023. “Roles of the Peroxisome Proliferator‐Activated Receptors (PPARs) in the Pathogenesis of Nonalcoholic Fatty Liver Disease (NAFLD).” Pharmacological Research 192: 106786. 10.1016/j.phrs.2023.106786 37146924

[97] Khan, Fahad , Deena Elsori , Meenakshi Verma , Shivam Pandey , Safia Obaidur Rab , Samra Siddiqui , Nadiyah M. Alabdallah , Mohd Saeed , and Pratibha Pandey . 2024. “Unraveling the Intricate Relationship Between Lipid Metabolism and Oncogenic Signaling Pathways.” Frontiers in Cell and Developmental Biology 12: 1399065. 10.3389/fcell.2024.1399065 PMC11199418 38933330

[98] Yohe, T. T. , B. D. Enger , L. Wang , H. L. M. Tucker , C. A. Ceh , C. L. M. Parsons , Z. Yu , and K. M. Daniels . 2018. “Short Communication: Does Early‐Life Administration of a Megasphaera Elsdenii Probiotic Affect Long‐Term Establishment of the Organism in the Rumen and Alter Rumen Metabolism in the Dairy Calf?” Journal of Dairy Science 101: 1747–1751. 10.3168/jds.2017-12551 29174148

[99] Zhang, Hao , Yuan Tian , Chunjie Xu , Miaomiao Chen , Zeyu Xiang , Lei Gu , Hanbing Xue , and Qing Xu . 2025. “Crosstalk Between Gut Microbiotas and Fatty Acid Metabolism in Colorectal Cancer.” Cell Death Discovery 11: 78. 10.1038/s41420-025-02364-5 PMC11865559 40011436

[100] Li, Liang , and Ying Ma . 2018. “The Effect of Soluble Saccharides on the Activity of Key Enzymes Linked to Methyl Ketone Synthesis in Lactococcus lactis.” CyTA ‐ Journal of Food 16: 325–330. 10.1080/19476337.2017.1401666

[101] Limsuwan, Tasanee , and Richard T. Lovell . 1981. “Intestinal Synthesis and Absorption of Vitamin B‐12 in Channel Catfish.” The Journal of Nutrition 111: 2125–2132. 10.1093/jn/111.12.2125 7310537

[102] Boran, Perran , Hatice Ezgi Baris , Eda Kepenekli , Can Erzik , Ahmet Soysal , and Duy M. Dinh . 2020. “The Impact of Vitamin B12 Deficiency on Infant Gut Microbiota.” European Journal of Pediatrics 179: 385–393. 10.1007/s00431-019-03517-2 31761973

[103] Franco‐Lopez, Julian , Melissa Duplessis , An Bui , Coralie Reymond , William Poisson , Lya Blais , Jasmine Chong , et al. 2020. “Correlations Between the Composition of the Bovine Microbiota and Vitamin b(12) Abundance.” Msystems 5: e00107–e00120. 10.1128/mSystems.00107-20 PMC7055655 32127420

[104] Sánchez, Cristina , Luis Franco , Patricia Regal , Alexandre Lamas , Alberto Cepeda , and Cristina Fente . 2021. “Breast Milk: A Source of Functional Compounds With Potential Application in Nutrition and Therapy.” Nutrients 13: 1026. 10.3390/nu13031026 PMC8005182 33810073

[105] Liu, Lily , Peifu Wu , Aiwei Guo , Yajin Yang , Fenfen Chen , and Qin Zhang . 2023. “Research Progress on the Regulation of Production Traits by Gastrointestinal Microbiota in Dairy Cows.” Frontiers in Veterinary Science 10: 1206346. 10.3389/fvets.2023.1206346 PMC10427726 37592942

[106] Wang, Yue , Yiguang Zhao , Xiangfang Tang , Xuemei Nan , Linshu Jiang , Hui Wang , Jun Liu , et al. 2024. “Nutrition, Gastrointestinal Microorganisms and Metabolites in Mastitis Occurrence and Control.” Animal Nutrition 17: 220–231. 10.1016/j.aninu.2024.01.010 PMC11126769 38800734

[107] Argov‐Argaman, Nurit , Miri Cohen‐Zinder , Haim Leibovich , Moran Yishay , Harel Eitam , Rotem Agmon , Oren Hadaya , et al. 2020. “Dietary Pomegranate Peel Improves Milk Quality of Lactating Ewes: Emphasis on Milk Fat Globule Membrane Properties and Antioxidative Traits.” Food Chemistry 313: 125822. 10.1016/j.foodchem.2019.125822 31931419

[108] Cao, Mingming , Liyun Bai , Haoyun Wei , Yantong Guo , Guodong Sun , Haoyang Sun , and Baoming Shi . 2024. “Dietary Supplementation with Pterostilbene Activates the PI3K‐AKT‐mTOR Signalling Pathway to Alleviate Progressive Oxidative Stress and Promote Placental Nutrient Transport.” Journal of Animal Science and Biotechnology 15: 133. 10.1186/s40104-024-01090-9 PMC11456245 39369257

[109] Alba, Davi F. , Karoline Leal , Marily H. Cunha , Gilneia da Rosa , Hiam Marcon , Carine F. Souza , Matheus D. Baldissera , et al. 2021. “Positive Effects of Biocholine Powder Dietary Supplementation on Milk Production and Quality, and Antioxidant Responses in Lactating Ewes: A New Nutritional Tool.” Heliyon 7: e06732. 10.1016/j.heliyon.2021.e06732 PMC8065297 33912712

[110] Fan, Yating , Jinglin Shen , Xinlu Liu , Junhao Cui , Jiayi Liu , Dongqiao Peng , and Yongcheng Jin . 2023. “β‐Sitosterol Suppresses Lipopolysaccharide‐Induced Inflammation and Lipogenesis Disorder in Bovine Mammary Epithelial Cells.” International Journal of Molecular Sciences 24: 14644. 10.3390/ijms241914644 PMC10572156 37834091

[111] Zhao, P. Y. , Z. F. Zhang , R. X. Lan , W. C. Liu , and I. H. Kim . 2017. “Effect of Lysophospholipids in Diets Differing in Fat Contents on Growth Performance, Nutrient Digestibility, Milk Composition and Litter Performance of Lactating Sows.” Animal 11: 984–990. 10.1017/S1751731116002238 27819219

[112] Wang, C. Q. , Y. S. Bai , X. Zhao , B. M. Shi , X. Y. Meng , and A. S. Shan . 2017. “Effects of Feeding Sodium stearoyl‐2‐lactylate Diets to Lactating Sows on Performance, Digestibility of Nutrients, Composition, and Fat Globule Size in Milk.” Journal of Animal Science 95: 5091–5099. 10.2527/jas2017.1851 PMC6292315 29293704

[113] Wang, Haitong , Xiaoli Ren , Li Liu , Zhuo Yang , Chunfang Li , Xiangnan Bao , Ayihumaer Amantuer , et al. 2025. “The Relationship between Protein Fraction Contents and Immune Cells in Milk.” Animals 15: 1578. 10.3390/ani15111578 PMC12153527 40509044

[114] She, Yuanhang , Jianying Liu , Minqiang Su , Yaokun Li , Yongqing Guo , Guangbin Liu , Ming Deng , et al. 2023. “A Study on Differential Biomarkers in the Milk of Holstein Cows With Different Somatic Cells Count Levels.” Animals 13: 2446. 10.3390/ani13152446 PMC10417570 37570255

[115] Mann, Elizabeth R. , Ying Ka Lam , and Holm H. Uhlig . 2024. “Short‐Chain Fatty Acids: Linking Diet, the Microbiome and Immunity.” Nature Reviews Immunology 24: 577–595. 10.1038/s41577-024-01014-8 38565643

[116] Wang, Lixuan , Weibo Jiang , Manshi Yang , Quezhu Danzeng , Shiyu Liu , and Mengying Cui . 2025. “Combination Strategies of Gut Microbiota in Cancer Therapy Through Metabolic Reprogramming and Immune Remodeling.” Cell Communication and Signaling 23: 270. 10.1186/s12964-025-02275-z PMC12139130 40474258

[117] Fassarella, Marina , Ellen E. Blaak , John Penders , Arjen Nauta , Hauke Smidt , and Erwin G. Zoetendal . 2021. “Gut Microbiome Stability and Resilience: Elucidating the Response to Perturbations in Order to Modulate Gut Health.” Gut 70: 595–605. 10.1136/gutjnl-2020-321747 33051190

[118] Nigam, Manisha , Kanchan Devi , Henrique D. M. Coutinho , and Abhay Prakash Mishra . 2024. “Exploration of Gut Microbiome and Inflammation: A Review on Key Signalling Pathways.” Cellular Signalling 118: 111140. 10.1016/j.cellsig.2024.111140 38492625

[119] Wu, Qiong , Ming‐Chao Liu , Jun Yang , Jiu‐Feng Wang , and Yao‐Hong Zhu . 2016. “Lactobacillus Rhamnosus GR‐1 Ameliorates Escherichia coli‐Induced Inflammation and Cell Damage Via Attenuation of ASC‐Independent NLRP3 Inflammasome Activation.” Applied and Environmental Microbiology 82: 1173–1182. 10.1128/AEM.03044-15 PMC4751844 26655757

[120] Wang, Yue , Xuemei Nan , Yiguang Zhao , Linshu Jiang , Hui Wang , Fan Zhang , Dengke Hua , et al. 2022. “Discrepancies Among Healthy, Subclinical Mastitic, and Clinical Mastitic Cows in Fecal Microbiome and Metabolome and Serum Metabolome.” Journal of Dairy Science 105: 7668–7688. 10.3168/jds.2021-21654 35931488

[121] Wen, Biyan , Yaoxing Huang , Guiqing Deng , Qingqing Yan , and Lin Jia . 2024. “Gut Microbiota Analysis and LC‐MS‐Based Metabolomics to Investigate AMPK/NF‐κB Regulated by Clostridium butyricum in the Treatment of Acute Pancreatitis.” Journal of Translational Medicine 22: 1072. 10.1186/s12967-024-05764-w PMC11600808 39604956

[122] Verma, Ankita , Sabra S. Inslicht , and Aditi Bhargava . 2024. “Gut‐Brain Axis: Role of Microbiome, Metabolomics, Hormones, and Stress in Mental Health Disorders.” Cells 13: 1436. 10.3390/cells13171436 PMC11394554 39273008

[123] Gieryńska, Małgorzata , Lidia Szulc‐Dąbrowska , Justyna Struzik , Matylda Barbara Mielcarska , and Karolina Paulina Gregorczyk‐Zboroch . 2022. “Integrity of the Intestinal Barrier: The Involvement of Epithelial Cells and Microbiota—A Mutual Relationship.” Animals 12: 145. 10.3390/ani12020145 PMC8772550 35049768

[124] Yu, Shunying , Yibin Sun , Xinyu Shao , Yuqing Zhou , Yang Yu , Xiaoyi Kuai , and Chunli Zhou . 2022. “Leaky Gut in Ibd: Intestinal Barrier‐Gut Microbiota Interaction.” Journal of Microbiology and Biotechnology 32: 825–834. 10.4014/jmb.2203.03022 PMC9628915 35791076

[125] Charoensappakit, Awirut , Kritsanawan Sae‐Khow , and Asada Leelahavanichkul . 2022. “Gut Barrier Damage and Gut Translocation of Pathogen Molecules in Lupus, an Impact of Innate Immunity (Macrophages and Neutrophils) in Autoimmune Disease.” International Journal of Molecular Sciences 23: 8223. 10.3390/ijms23158223 PMC9367802 35897790

[126] Xiang, Xing‐Wei , Rui Wang , Li‐Wen Yao , Yu‐Fang Zhou , Pei‐Long Sun , Bin Zheng , and Yu‐Feng Chen . 2021. “Anti‐Inflammatory Effects of Mytilus Coruscus Polysaccharide on raw264.7 Cells and Dss‐Induced Colitis in Mice.” Marine Drugs 19: 468. 10.3390/md19080468 PMC8400803 34436307

[127] Zhang, Jinning , Wenlong Li , Yongjie Tang , Xueqin Liu , Hailiang Zhang , Yueling Zhou , Yachun Wang , Wei Xiao , and Ying Yu . 2022. “Testing Two Somatic Cell Count Cutoff Values for Bovine Subclinical Mastitis Detection Based on Milk Microbiota and Peripheral Blood Leukocyte Transcriptome Profile.” Animals (Basel) 12: 1694. 10.3390/ani12131694 PMC9264859 35804592

[128] Jiang, Liqiang , Jingjing Wang , Ziyi Liu , Aimin Jiang , Shuangqiu Li , Di Wu , Yong Zhang , et al. 2020. “Sodium Butyrate Alleviates Lipopolysaccharide‐Induced Inflammatory Responses by Down‐Regulation of NF‐κB, NLRP3 Signaling Pathway, and Activating Histone Acetylation in Bovine Macrophages.” Frontiers in Veterinary Science 7: 579674. 10.3389/fvets.2020.579674 PMC7674777 33251265

[129] Bennett, S. , I. Fliss , L. Ben Said , F. Malouin , and P. Lacasse . 2022. “Efficacy of Bacteriocin‐Based Formula for Reducing Staphylococci, Streptococci, and Total Bacterial Counts on Teat Skin of Dairy Cows.” Journal of Dairy Science 105: 4498–4507. 10.3168/jds.2021-21381 35346465

[130] Nagahata, Hajime , Takuma Mukai , Yo Natsume , Miyuki Okuda , Tatsuya Ando , Keiichi Hisaeda , Satoshi Gondaira , and Hidetoshi Higuchi . 2020. “Effects of Intramammary Infusion of Bifidobacterium breve on Mastitis Pathogens and Somatic Cell Response in Quarters from Dairy Cows With Chronic Subclinical Mastitis.” Animal Science Journal 91: e13406. 10.1111/asj.13406 32578314

[131] Qiu, Min , Lianjun Feng , Caijun Zhao , Siyuan Gao , Lijuan Bao , Yihong Zhao , Yunhe Fu , and Xiaoyu Hu . 2022. “Commensal Bacillus Subtilis From Cow Milk Inhibits Staphylococcus Aureus Biofilm Formation and Mastitis in Mice.” Fems Microbiology Ecology 98: fiac065. 10.1093/femsec/fiac065 35648454

[132] Bennett, Samantha , Laila Ben Said , Pierre Lacasse , François Malouin , and Ismail Fliss . 2021. “Susceptibility to Nisin, Bactofencin, Pediocin and Reuterin of Multidrug Resistant Staphylococcus Aureus, Streptococcus Dysgalactiae and Streptococcus Uberis Causing Bovine Mastitis.” Antibiotics 10: 1418. 10.3390/antibiotics10111418 PMC8614789 34827356

[133] Luo, Shuangyan , Yuxia Wang , Xinyun Kang , Panpan Liu , and Guiqin Wang . 2022. “Research Progress on the Association between Mastitis and Gastrointestinal Microbes in Dairy Cows and the Effect of Probiotics.” Microbial Pathogenesis 173: 105809. 10.1016/j.micpath.2022.105809 36183956

[134] Xu, Haiyan , Weiqiang Huang , Qiangchuan Hou , Lai‐Yu Kwok , Zhihong Sun , Huimin Ma , Feiyan Zhao , Yuan‐Kun Lee , and Heping Zhang . 2017. “The Effects of Probiotics Administration on the Milk Production, Milk Components and Fecal Bacteria Microbiota of Dairy Cows.” Science Bulletin 62: 767–774. 10.1016/j.scib.2017.04.019 36659272

[135] Wang, Yue , Xuemei Nan , Yiguang Zhao , Linshu Jiang , Hui Wang , Fan Zhang , Dengke Hua , et al. 2021. “Dietary Supplementation of Inulin Ameliorates Subclinical Mastitis Via Regulation of Rumen Microbial Community and Metabolites in Dairy Cows.” Microbiology Spectrum 9: e0010521. 10.1128/Spectrum.00105-21 PMC8557905 34494854

[136] Aabdin, Zain Ul , Muhammad Shahid Bilal , Hongyu Dai , Juma Ahamed Abaker , Xinxin Liu , Sahito Benazir , Jinyu Yan , and Xiangzhen Shen . 2018. “NOD1/NF‐κB Signaling Pathway Inhibited by Sodium Butyrate in the Mammary Gland of Lactating Goats During Sub‐Acute Ruminal Acidosis.” Microbial Pathogenesis 122: 58–62. 10.1016/j.micpath.2018.05.045 29857120

[137] Jiménez, Esther , Susana Manzano , Dietmar Schlembach , Krzysztof Arciszewski , Rocio Martin , Kaouther Ben Amor , Mieke Roelofs , et al. 2021. “Ligilactobacillus salivarius PS2 Supplementation During Pregnancy and Lactation Prevents Mastitis: A Randomised Controlled Trial.” Microorganisms 9: 1933. 10.3390/microorganisms9091933 PMC8469334 34576828

[138] Urakawa, Megumi , Tao Zhuang , Hidetoshi Sato , Satoru Takanashi , Kozue Yoshimura , Yuma Endo , Teppei Katsura , et al. 2022. “Prevention of Mastitis in Multiparous Dairy Cows With a Previous History of Mastitis by Oral Feeding with Probiotic Bacillus Subtilis.” Animal Science Journal 93: e13764. 10.1111/asj.13764 PMC9541589 36085592

[139] Li, Kefei , Xin Ran , Yiruo Zeng , Shubo Li , Guiqiu Hu , Xiaoxuan Wang , Ying Li , et al. 2023. “Maslinic Acid Alleviates LPS‐Induced Mice Mastitis by Inhibiting Inflammatory Response, Maintaining the Integrity of the Blood‐Milk Barrier and Regulating Intestinal Flora.” International Immunopharmacology 122: 110551. 10.1016/j.intimp.2023.110551 37406397

[140] Li, Kefei , Xin Ran , Jiaxi Han , Huiping Ding , Xiaoxuan Wang , Yutao Li , Weiwei Guo , et al. 2025. “Astragalus Polysaccharide Alleviates Mastitis Disrupted by Staphylococcus Aureus Infection by Regulating Gut Microbiota and SCFAs Metabolism.” International Journal of Biological Macromolecules 286: 138422. 10.1016/j.ijbiomac.2024.138422 39647752

[141] Yu, Jie , Weicheng Li , Ruibo Xu , Xiaoye Liu , Guangqi Gao , Lai‐Yu Kwok , Yongfu Chen , et al. 2024. “Probio‐M9, a Breast Milk‐Originated Probiotic, Alleviates Mastitis and Enhances Antibiotic Efficacy: Insights into the Gut‐Mammary Axis.” iMeta 3: e224. 10.1002/imt2.224 PMC11316926 39135694

[142] Džermeikaitė, Karina , Dovilė Bačėninaitė , and Ramūnas Antanaitis . 2023. “Innovations in Cattle Farming: Application of Innovative Technologies and Sensors in the Diagnosis of Diseases.” Animals 13: 780. 10.3390/ani13050780 PMC10000156 36899637

[143] Costa, A. , A. Goi , M. Penasa , G. Nardino , L. Posenato , and M. De Marchi . 2021. “Variation of Immunoglobulins G, A, and M and Bovine Serum Albumin Concentration in Holstein Cow Colostrum.” Animal 15: 100299. 10.1016/j.animal.2021.100299 34167023

[144] Dawod, Bassel , Jean S. Marshall , and Meghan B. Azad . 2021. “Breastfeeding and the Developmental Origins of Mucosal Immunity: How Human Milk Shapes the Innate and Adaptive Mucosal Immune Systems.” Current Opinion in Gastroenterology 37: 547–556. 10.1097/MOG.0000000000000778 PMC11451935 34634003

[145] Diebolder, Christoph A. , Frank J. Beurskens , Rob N. de Jong , Roman I. Koning , Kristin Strumane , Margaret A. Lindorfer , Marleen Voorhorst , et al. 2014. “Complement Is Activated by IgG Hexamers Assembled at the Cell Surface.” Science 343: 1260–1263. 10.1126/science.1248943 PMC4250092 24626930

[146] Donald, Katherine , Antonio Serapio‐Palacios , Zachary Gerbec , Tahereh Bozorgmehr , Ravi Holani , Ana Raquel Cruz , Pamela Schnupf , and B. Brett Finlay . 2024. “Secretory IgA in Breast Milk Protects Against Asthma Through Modulation of the Gut Microbiota.” Cell Reports 43: 114835. 10.1016/j.celrep.2024.114835 39368092

[147] Ragland, Stephanie A. , and Alison K. Criss . 2017. “From Bacterial Killing to Immune Modulation: Recent Insights into the Functions of Lysozyme.” Plos Pathogens 13: e1006512. 10.1371/journal.ppat.1006512 PMC5608400 28934357

[148] Eker, Furkan , Emir Akdaşçi , Hatice Duman , Yalçın Mert Yalçıntaş , Ahmet Alperen Canbolat , Arda Erkan Kalkan , Sercan Karav , and Dunja Šamec . 2024. “Antimicrobial Properties of Colostrum and Milk.” Antibiotics 13: 251. 10.3390/antibiotics13030251 PMC10967647 38534686

[149] Li, Chuangang , Yanan Sun , Tingchao He , Yao Lu , Ignatius Man‐Yau Szeto , Sufang Duan , Yifan Zhang , et al. 2023. “Synergistic Effect of Lactoferrin and Osteopontin on Intestinal Barrier Injury.” International Journal of Biological Macromolecules 253: 127416. 10.1016/j.ijbiomac.2023.127416 37838132

[150] Tang, Xiaopeng , Hu Liu , Shufen Yang , Zuohua Li , Jinfeng Zhong , and Rejun Fang . 2016. “Epidermal Growth Factor and Intestinal Barrier Function.” Mediators of Inflammation 2016: 1927348. 10.1155/2016/1927348 PMC4976184 27524860

[151] Zheng, Yu , Yongli Song , Qi Han , Wenjie Liu , Jiuzhi Xu , Zhengquan Yu , Ran Zhang , and Ning Li . 2018. “Intestinal Epithelial Cell‐Specific IGF1 Promotes the Expansion of Intestinal Stem Cells During Epithelial Regeneration and Functions on the Intestinal Immune Homeostasis.” American Journal of Physiology‐Endocrinology and Metabolism 315: E638–E649. 10.1152/ajpendo.00022.2018 29783855

[152] Kawathe, Radhika , Vykuntaraju K. Gowda , Viveka S. Reddy , Annsmol P. Markose , Uddhava V. Kinhal , and Kannan Rangiah . 2025. “Mass Spectrometry Analysis of B Vitamins in a Drop of Maternal Milk with Concern for Child Health.” Microchemical Journal 212: 113556. 10.1016/j.microc.2025.113556

[153] Yadav, Dhawal Kant , Yallappa M. Somagond , Pravasini Das , Surender Singh Lathwal , Aarti Kamboj , Mohanned Naif Alhussien , and Ajay Kumar Dang . 2024. “Injection of Antioxidant Trace Minerals/Vitamins into Peripartum Crossbred Cows Improves the Nutritional and Immunological Properties of Colostrum/Milk and the Health of Their Calves Under Heat Stress Conditions.” Tropical Animal Health and Production 56: 225. 10.1007/s11250-024-04084-x 39066797

[154] Natividad, Jane M. , Andreas Rytz , Sonia Keddani , Gabriela Bergonzelli , and Clara L. Garcia‐Rodenas . 2020. “Blends of Human Milk Oligosaccharides Confer Intestinal Epithelial Barrier Protection in Vitro.” Nutrients 12: 3047. 10.3390/nu12103047 PMC7599875 33027993

[155] Hegar, Badriul , Yulianti Wibowo , Ray Wagiu Basrowi , Reza Gunadi Ranuh , Subianto Marto Sudarmo , Zakiudin Munasir , Alpha Fardah Atthiyah , et al. 2019. “The Role of Two Human Milk Oligosaccharides, 2′‐Fucosyllactose and Lacto‐N‐Neotetraose, in Infant Nutrition.” Pediatric Gastroenterology, Hepatology & Nutrition 22: 330–340. 10.5223/pghn.2019.22.4.330 PMC6629589 31338308

[156] Torres‐Castro, Paulina , Mar Abril‐Gil , María J Rodríguez‐Lagunas , Margarida Castell , Francisco J. Pérez‐Cano , and Àngels Franch . 2018. “Tgf‐β2, egf, and fgf21 Growth Factors Present in Breast Milk Promote Mesenteric Lymph Node Lymphocytes Maturation in Suckling Rats.” Nutrients 10: 1171. 10.3390/nu10091171 PMC6163676 30150532

[157] Torres‐Castro, Paulina , Blanca Grases‐Pintó , Mar Abril‐Gil , Margarida Castell , María J Rodríguez‐Lagunas , Francisco J. Pérez‐Cano , and Àngels Franch . 2020. “Modulation of the Systemic Immune Response in Suckling Rats by Breast Milk tgf‐β2, egf and fgf21 Supplementation.” Nutrients 12: 1888. 10.3390/nu12061888 PMC7353385 32599899

[158] Zhang, Siqi , Qingyuan Yu , Yukun Sun , Guangning Zhang , Yonggen Zhang , and Hangshu Xin . 2025. “Alleviating the Effect of Branched‐Chain Fatty Acids on the Lipopolysaccharide‐Induced Inflammatory Response in Calf Small Intestinal Epithelial Cells.” Antioxidants 14: 608. 10.3390/antiox14050608 PMC12109260 40427489

[159] Ramiro‐Cortijo, David , Pratibha Singh , Yan Liu , Esli Medina‐Morales , William Yakah , Steven D. Freedman , and Camilia R. Martin . 2020. “Breast Milk Lipids and Fatty Acids in Regulating Neonatal Intestinal Development and Protecting Against Intestinal Injury.” Nutrients 12: 534. 10.3390/nu12020534 PMC7071444 32092925

[160] Liu, Jia , Bo Li , Carol Lee , Haitao Zhu , Shan Zheng , and Agostino Pierro . 2019. “Protective Effects of Lactoferrin on Injured Intestinal Epithelial Cells.” Journal of Pediatric Surgery 54: 2509–2513. 10.1016/j.jpedsurg.2019.08.046 31668400

[161] Koch, Meghan A. , Gabrielle L. Reiner , Kyler A. Lugo , Lieselotte S. M. Kreuk , Alison G. Stanbery , Eduard Ansaldo , Thaddeus D. Seher , William B. Ludington , and Gregory M. Barton . 2016. “Maternal IgG and IgG Antibodies Dampen Mucosal T Helper Cell Responses in Early Life.” Cell 165: 827–841. 10.1016/j.cell.2016.04.055 PMC4866587 27153495

[162] Guo, Wenli , Shuai Liu , Muhammad Z. Khan , Jingjun Wang , Tianyu Chen , Gibson M. Alugongo , Shengli Li , and Zhijun Cao . 2024. “Bovine Milk Microbiota: Key Players, Origins, and Potential Contributions to Early‐Life Gut Development.” Journal of Advanced Research 59: 49–64. 10.1016/j.jare.2023.06.016 PMC11081965 37423549

[163] Thai, Julie D. , and Katherine E. Gregory . 2020. “Bioactive Factors in Human Breast Milk Attenuate Intestinal Inflammation During Early Life.” Nutrients 12: 581. 10.3390/nu12020581 PMC7071406 32102231

[164] Morrin, Sinead T. , Rachael H. Buck , Michael Farrow , and Rita M. Hickey . 2021. “Milk‐Derived Anti‐Infectives and Their Potential to Combat Bacterial and Viral Infection.” Journal of Functional Foods 81: 104442. 10.1016/j.jff.2021.104442

[165] Kalbermatter, Cristina , Nerea Fernandez Trigo , Sandro Christensen , and Stephanie C. Ganal‐Vonarburg . 2021. “Maternal Microbiota, Early Life Colonization and Breast Milk Drive Immune Development in the Newborn.” Frontiers in Immunology 12: 683022. 10.3389/fimmu.2021.683022 PMC8158941 34054875

[166] Wang, Yanli , Yiyao Gong , Muhammad Salman Farid , and Changhui Zhao . 2024. “Milk: A Natural Guardian for the Gut Barrier.” Journal of Agricultural and Food Chemistry 72: 8285–8303. 10.1021/acs.jafc.3c06861 38588092

[167] Vega‐Bautista, Alan , Mireya de la Garza , Julio César Carrero , Rafael Campos‐Rodríguez , Marycarmen Godínez‐Victoria , and Maria Elisa Drago‐Serrano . 2019. “The Impact of Lactoferrin on the Growth of Intestinal Inhabitant Bacteria.” International Journal of Molecular Sciences 20: 4707. 10.3390/ijms20194707 PMC6801499 31547574

[168] Azagra‐Boronat, Ignasi , Alba Tres , Malén Massot‐Cladera , Àngels Franch , Margarida Castell , Francesc Guardiola , Francisco J. Pérez‐Cano , and Maria J. Rodríguez‐Lagunas . 2020. “Lactobacillus fermentum CECT5716 Supplementation in Rats during Pregnancy and Lactation Impacts Maternal and Offspring Lipid Profile, Immune System and Microbiota.” Cells 9: 575. 10.3390/cells9030575 PMC7140451 32121244

[169] Zhong, Zhi , Hai Tang , Tingting Shen , Xinwei Ma , Feiyan Zhao , Lai‐Yu Kwok , Zhihong Sun , Menghe Bilige , and Heping Zhang . 2022. “Bifidobacterium Animalis Subsp. Lactis Probio‐M8 Undergoes Host Adaptive Evolution by Glcu Mutation and Translocates to the Infant's Gut Via Oral‐/Entero‐Mammary Routes Through Lactation.” Microbiome 10: 197. 10.1186/s40168-022-01398-6 PMC9682673 36419187

[170] Xie, Qinggang , Dongying Cui , Qinchao Zhu , Xuewen Qin , Daxi Ren , and Xiaoxi Xu . 2023. “Supplementing Maternal Diet with Milk Oligosaccharides and Probiotics Helps Develop the Immune System and Intestinal Flora of Offsprings.” Food Science & Nutrition 11: 6868–6877. 10.1002/fsn3.3579 PMC10630837 37970377

[171] Alemu, Bekalu Kassie , Getnet Gedefaw Azeze , Ling Wu , So Ling Lau , Chi Chiu Wang , and Yao Wang . 2023. “Effects of Maternal Probiotic Supplementation on Breast Milk Microbiome and Infant Gut Microbiome and Health: A Systematic Review and Meta‐Analysis of Randomized Controlled Trials.” American Journal of Obstetrics & Gynecology MFM 5: 101148. 10.1016/j.ajogmf.2023.101148 37660760

[172] Wang, Gang , Xinyu Wang , Yonghang Ma , Shuang Cai , Lijie Yang , Yuxin Fan , Xiangfang Zeng , and Shiyan Qiao . 2022. “Lactobacillus Reuteri Improves the Development and Maturation of Fecal Microbiota in Piglets Through Mother‐to‐Infant Microbe and Metabolite Vertical Transmission.” Microbiome 10: 211. 10.1186/s40168-022-01336-6 PMC9717520 36461096

[173] Donovan, Sharon M . 2016. “The Role of Lactoferrin in Gastrointestinal and Immune Development and Function: A Preclinical Perspective.” The Journal of Pediatrics 173(Suppl): S16–S28. 10.1016/j.jpeds.2016.02.072 27234407

[174] Jahan, Marefa , Susie Kracht , Yen Ho , Ziaul Haque , Birendra N. Bhattachatyya , Peter C. Wynn , and Bing Wang . 2017. “Dietary Lactoferrin Supplementation to Gilts During Gestation and Lactation Improves Pig Production and Immunity.” PLoS One 12: e0185817. 10.1371/journal.pone.0185817 PMC5638254 29023467

[175] Rodríguez, Juan M. , Leónides Fernández , and Valerie Verhasselt . 2021. “The Gut–Breast Axis: Programming Health for Life.” Nutrients 13: 606. 10.3390/nu13020606 PMC7917897 33673254

[176] Marino, Maria Michela , Bianca Maria Nastri , Marina D'Agostino , Rossella Risolo , Alessandra De Angelis , Giuliana Settembre , Monica Rienzo , et al. 2022. “Does Gut‐Breast Microbiota Axis Orchestrates Cancer Progression?” Endocrine, Metabolic & Immune Disorders ‐ Drug Targets 22: 1111–1122. 10.2174/1871530322666220331145816 35362389

[177] Ibrahim, Nour , Nawal Alsadi , Hamed Yasavoli‐Sharahi , Roghayeh Shahbazi , Mary Joe Hebbo , Darshan Kambli , Florencia Balcells , and Chantal Matar . 2025. “Berberine Inhibits Breast Cancer Stem Cell Development and Decreases Inflammation: Involvement of miRNAs and il‐6.” Current Developments in Nutrition 9: 104532. 10.1016/j.cdnut.2024.104532 PMC11786844 39896297

[178] Ivanova, E. , C. Hue‐Beauvais , A. Chaulot‐Talmon , J. Castille , J. Laubier , C. De Casanove , A. Aubert‐Frambourg , et al. 2023. “DNA Methylation and Gene Expression Changes in Mouse Mammary Tissue During Successive Lactations: Part I ‐ The Impact of Inflammation.” Epigenetics 18: 2215633. 10.1080/15592294.2023.2215633 PMC10732689 37302099

[179] Xue, Qiuyun , Yurong Huang , Chenglong Cheng , Yuting Wang , Faxue Liao , Qiangjun Duan , Xiao Wang , and Chenggui Miao . 2023. “Progress in Epigenetic Regulation of Milk Synthesis, With Particular Emphasis on mRNA Regulation and DNA Methylation.” Cell Cycle 22: 1675–1693. 10.1080/15384101.2023.2225939 PMC10446801 37409592

[180] Kwa, Maryann , Claudia S. Plottel , Martin J. Blaser , and Sylvia Adams . 2016. “The Intestinal Microbiome and Estrogen Receptor‐Positive Female Breast Cancer.” Journal of the National Cancer Institute 108: djw026. 10.1093/jnci/djw029 PMC5017946 27107051

[181] He, Jin , Peiwen Zhang , Linyuan Shen , Lili Niu , Ya Tan , Lei Chen , Ye Zhao , et al. 2020. “Short‐Chain Fatty Acids and Their Association With Signalling Pathways in Inflammation, Glucose and Lipid Metabolism.” International Journal of Molecular Sciences 21: 6356. 10.3390/ijms21176356 PMC7503625 32887215

[182] Wu, Yongjiang , Jingbo Chen , Yawang Sun , Xianwen Dong , Zili Wang , Juncai Chen , and Guozhong Dong . 2020. “PGN and lta From Staphylococcus Aureus Induced Inflammation and Decreased Lactation through Regulating Dna Methylation and Histone h3 Acetylation in Bovine Mammary Epithelial Cells.” Toxins 12: 238. 10.3390/toxins12040238 PMC7232188 32283626

[183] Chen, Jingbo , Yongjiang Wu , Yawang Sun , Xianwen Dong , Zili Wang , Zhu Zhang , Yanli Xiao , and Guozhong Dong . 2019. “Bacterial Lipopolysaccharide Induced Alterations of Genome‐Wide DNA Methylation and Promoter Methylation of Lactation‐Related Genes in Bovine Mammary Epithelial Cells.” Toxins 11: 298. 10.3390/toxins11050298 PMC6563294 31137708

[184] Pärnänen, Katariina , Antti Karkman , Jenni Hultman , Christina Lyra , Johan Bengtsson‐Palme , D G Joakim Larsson , Samuli Rautava , et al. 2018. “Maternal Gut and Breast Milk Microbiota Affect Infant Gut Antibiotic Resistome and Mobile Genetic Elements.” Nature Communications 9: 3891. 10.1038/s41467-018-06393-w PMC6155145 30250208

[185] Gialeli, Giannoula , Ourania Panagopoulou , Georgios Liosis , and Tania Siahanidou . 2023. “Potential Epigenetic Effects of Human Milk on Infants' Neurodevelopment.” Nutrients 15: 3614. 10.3390/nu15163614 PMC10460013 37630804

[186] Zhang, Weiqin , Yong Zhang , Yalin Li , Da Ma , Heping Zhang , and Lai‐Yu Kwok . 2022. “Lacticaseibacillus rhamnosus Probio‐M9‐Driven Mouse Mammary Tumor‐Inhibitory Effect Is Accompanied by Modulation of Host Gut Microbiota, Immunity, and Serum Metabolome.” Nutrients 15: 5. 10.3390/nu15010005 PMC9824041 36615662

[187] Jiang, Yue , and Yuanyuan Li . 2024. “Nutrition Intervention and Microbiome Modulation in the Management of Breast Cancer.” Nutrients 16: 2644. 10.3390/nu16162644 PMC11356826 39203781

[188] Wang, Xinmeng , Qian Geng , Hua Jiang , Jingyan Yue , Chunjian Qi , and Lanqun Qin . 2025. “Fecal Microbiota Transplantation Enhanced the Effect of Chemoimmunotherapy by Restoring Intestinal Microbiota in LLC Tumor‐Bearing Mice.” Bmc Immunol 26: 30. 10.1186/s12865-025-00710-x PMC11978186 40200137

[189] Parida, Sheetal , and Dipali Sharma . 2019. “The Microbiome‐Estrogen Connection and Breast Cancer Risk.” Cells 8: 1642. 10.3390/cells8121642 PMC6952974 31847455

[190] Zou, Yao , and Chong‐Xuan Han . 2020. “Interaction Between Intestinal Microorganisms and Carbohydrates of Mammals and Its Influence.” The Journal of Applied Ecology 31: 3959–3968. 10.13287/j.1001-9332.202011.037 33300747

[191] Zhao, Caijun , Keyi Wu , Haoyang Hao , Yihong Zhao , Lijuan Bao , Min Qiu , Yuhong He , et al. 2023. “Gut Microbiota‐Mediated Secondary Bile Acid Alleviates Staphylococcus Aureus‐Induced Mastitis Through the TGR5‐cAMP‐PKA‐NF‐κB/NLRP3 Pathways in Mice.” Npj Biofilms and Microbiomes 9: 8. 10.1038/s41522-023-00374-8 PMC9908919 36755021

[192] Yu, Zhuo‐ping , Dong‐dong Xu , Lai‐feng Lu , Xiao‐dong Zheng , and Wei Chen . 2016. “Immunomodulatory Effect of a Formula Developed From American Ginseng and Chinese Jujube Extracts in Mice.” Journal of Zhejiang University‐Science B 17: 147–157. 10.1631/jzus.B1500170 PMC4757584 26834015

[193] Di, Zhang , and Li Jingnan . 2020. “Advances in the Role of Intestinal Microbiota in Viral Infectious Diseases.” Chinese Journal of Internal Medicine 59: 477–480. 10.3760/cma.j.cn112138-20200227-00146 32174097

[194] Wu, Li , Liu‐qin He , Zhi‐jie Cui , Gang Liu , Kang Yao , Fei Wu , Jun Li , and Tie‐jun Li . 2015. “Effects of Reducing Dietary Protein on the Expression of Nutrition Sensing Genes (Amino Acid Transporters) in Weaned Piglets.” Journal of Zhejiang University‐Science B 16: 496–502. 10.1631/jzus.B1400259 PMC4471601 26055911

[195] Brito, Luiz F. , Hinayah R. Oliveira , Betty R. McConn , Allan P. Schinckel , Aitor Arrazola , Jeremy N. Marchant‐Forde , and Jay S. Johnson . 2020. “Large‐Scale Phenotyping of Livestock Welfare in Commercial Production Systems: A New Frontier in Animal Breeding.” Frontiers in Genetics 11: 793. 10.3389/fgene.2020.00793 PMC7411239 32849798

[196] Qi, Xin , Donghui Yan , Jiachen Zuo , Rui Wang , and Jiajia Chen . 2021. “Development of a Novel Chemokine Signaling‐Based Multigene Signature to Predict Prognosis and Therapeutic Response in Colorectal Cancer.” Journal of Zhejiang University‐Science B 22: 1053–1059. 10.1631/jzus.B2100412 PMC8669326 34904417

[197] Hu, Xiaoyu , Shumin Li , Yunhe Fu , and Naisheng Zhang . 2019. “Targeting Gut Microbiota As a Possible Therapy for Mastitis.” European Journal of Clinical Microbiology & Infectious Diseases Official Publication of the European Society of Clinical Microbiology 38: 1409–1423. 10.1007/s10096-019-03549-4 31079312

[198] de Andrés, Javier , Esther Jiménez , Irene Espinosa‐Martos , Juan Miguel Rodríguez , and María‐Teresa García‐Conesa . 2018. “An Exploratory Search for Potential Molecular Targets Responsive to the Probiotic Lactobacillus Salivarius ps2 in Women With Mastitis: Gene Expression Profiling Vs. Interindividual Variability.” Frontiers in Microbiology 9: 2166. 10.3389/fmicb.2018.02166 PMC6146105 30271395

[199] Kong, Fanlin , Shuo Wang , Yijia Zhang , Chen Li , Dongwen Dai , Yajing Wang , Zhijun Cao , et al. 2025. “Alanine Derived from Ruminococcus_E Bovis Alleviates Energy Metabolic Disorders During the Peripartum Period by Providing Glucogenic Precursors.” Research 8: 0682. 10.34133/research.0682 PMC12022398 40290137

[200] Kong, Fanlin , Shuo Wang , Yijia Zhang , Chen Li , Dongwen Dai , Cheng Guo , Yajing Wang , et al. 2025. “Rumen Microbiome Associates with Postpartum Ketosis Development in Dairy Cows: A Prospective Nested Case‐Control Study.” Microbiome 13: 69. 10.1186/s40168-025-02072-3 PMC11889851 40057813

[201] Zhong, Pei , Qin Li , Yanmei Zhang , Cheng Guo , Mahmoud M. Abdelsattar , and Yanliang Bi . 2025. “Precision Microbial Regulation: Strategies for Modulating GIT Microbiota for Host Health.” iMetaOmics 2: e54. 10.1002/imo2.54 PMC12806292 41675700

